# Dynamic Manipulation of Droplets on Liquid-Infused Surfaces Using Photoresponsive Surfactant

**DOI:** 10.1021/acscentsci.3c00982

**Published:** 2024-02-27

**Authors:** Xichen Liang, Kseniia M. Karnaukh, Lei Zhao, Serena Seshadri, Austin J. DuBose, Sophia J. Bailey, Qixuan Cao, Marielle Cooper, Hao Xu, Michael Haggmark, Matthew E. Helgeson, Michael Gordon, Paolo Luzzatto-Fegiz, Javier Read de Alaniz, Yangying Zhu

**Affiliations:** † Department of Chemical Engineering, University of California at Santa Barbara, Santa Barbara, California 93106-5070, United States; ‡ Department of Chemistry, University of California at Santa Barbara, Santa Barbara, California 93106-5070, United States; § Department of Mechanical Engineering, University of California at Santa Barbara, Santa Barbara, California 93106-5070, United States; ∥ Department of Physics, University of California at Santa Barbara, Santa Barbara, California 93106-5070, United States

## Abstract

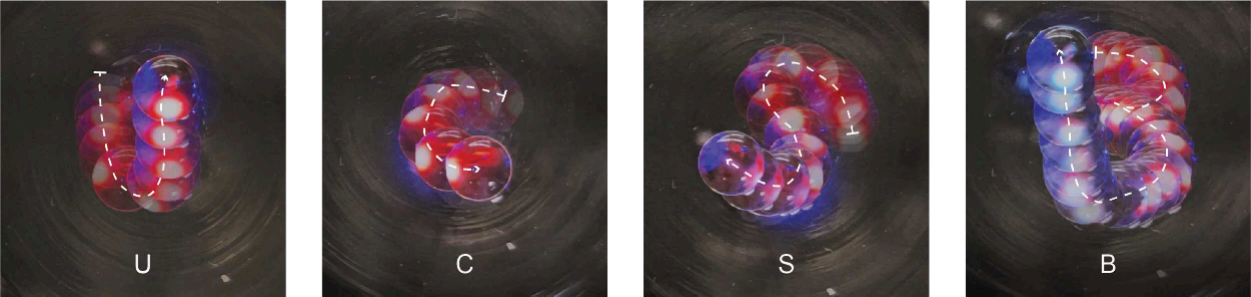

Fast and programmable transport of droplets on a substrate is desirable in microfluidic, thermal, biomedical, and energy devices. Photoresponsive surfactants are promising candidates to manipulate droplet motion due to their ability to modify interfacial tension and generate “photo-Marangoni” flow under light stimuli. Previous works have demonstrated photo-Marangoni droplet migration in liquid media; however, migration on other substrates, including solid and liquid-infused surfaces (LIS), remains an outstanding challenge. Moreover, models of photo-Marangoni migration are still needed to identify optimal photoswitches and assess the feasibility of new applications. In this work, we demonstrate 2D droplet motion on liquid surfaces and on LIS, as well as rectilinear motion in solid capillary tubes. We synthesize photoswitches based on spiropyran and merocyanine, capable of tension changes of up to 5.5 mN/m across time scales as short as 1.7 s. A millimeter-sized droplet migrates at up to 5.5 mm/s on a liquid, and 0.25 mm/s on LIS. We observe an optimal droplet size for fast migration, which we explain by developing a scaling model. The model also predicts that faster migration is enabled by surfactants that maximize the ratio between the tension change and the photoswitching time. To better understand migration on LIS, we visualize the droplet flow using tracer particles, and we develop corresponding numerical simulations, finding reasonable agreement. The methods and insights demonstrated in this study enable advances for manipulation of droplets for microfluidic, thermal and water harvesting devices.

## Abstract 2

We demonstrate real-time manipulation of droplet motion on liquid-infused surfaces, in solid-wall channels, and on liquids using novel ionic and nonionic photoresponsive surfactants.

## Introduction

Manipulating droplets on surfaces and interfaces is critical for the efficiency and tunability of water desalination,^1^ condensation,^2−5^ liquid transport in microfluidics,^6^ and digital bioassays.^7^ For example, condensation, a key process in power generation and thermal management, requires fast removal of droplets from the substrates for efficient heat transfer.^4,8^ Passive methods to transport droplets on surfaces have focused on engineering asymmetric surface structures^9−18^ and gradient chemistry.^19−22^ However, these surfaces typically require complex fabrication and do not offer real-time control capability. Active methods that can dynamically tune droplet motions have been developed using electric,^6,23−25^ magnetic,^26−29^ acoustic,^30^ chemical,^31,32^ thermal,^33,34^ and photothermal^35,36^ stimuli. While electric, magnetic, and photothermal systems may require a high external field to actuate droplet motion, thermal systems can have a slow response. To realize spatial control, some of these methods require patterning of microstructures.^37^

Manipulation methods based purely on light stimuli are attractive as light offers spatial resolution down to the diffraction limit, can be easily reconfigured, and does not require the fabrication of microstructures such as electrode arrays.^38^ Several studies have investigated photosensitive substrates such as TiO_2_ or ZnO.^39,40^ Although the contact angle of water on these surfaces can decrease by 70–100° upon UV illumination, direct manipulation of droplet movement has not been reported. Optical tweezers based on the transfer of photon momentum have been extensively studied to trap and manipulate solid microparticles.^41^ However, they have also not been used to control liquid droplets directly.

Alternatively, photoresponsive surfactants have recently been used to manipulate multiphase fluid systems. These surfactants can change their molecular conformation when illuminated by light with an appropriate wavelength. When integrated with polymer film and attached to a surface, these photoresponsive surfactants can modify the wettability of the surface by adjusting the irradiation condition.^42,43^ When dissolved in a liquid, these surfactants can change the osmotic pressure of the fluid, which can be used to control particle motion.^44,45^ In addition, the photoswitch of surfactants induces a local change of the surface tension or interfacial tension, which can further generate a Marangoni flow. Compared to photothermal effects (i.e., using light to heat fluid for liquid motion based on Marangoni-flow or temperature-dependent surface tension force), photoresponsive surfactants require a significantly lower light intensity to generate a comparable surface tension change,^46^ and no light-absorbing substrates or nanoparticle additives are needed.

Using photoresponsive surfactants, including azobenzene containing trimethyl-ammonium bromide (AzoTAB), donor–acceptor Stenhouse adduct (DASA), and spiropyrans,^47,48^ recent works have demonstrated dynamically reconfigurable emulsions,^49−51^ trapping and manipulation of solid objects,^52^ controlling particle deposition on surfaces,^53^ moving droplets immersed in^47,54,55^ and on the surface of an immiscible liquid,^47,56,57^ and bubble departure from solid surfaces.^46^ While substantial progress has been made in manipulating droplets in liquid media, the movement of liquid droplets on solid or liquid-infused surfaces using the photo-Marangoni effect has not been achieved so far. This is particularly important for practical applications involving liquid–solid interactions, including condensation for thermal, power, and water harvesting systems,^4,20^ chemical microreactors,^58,59^ biosciences technologies,^60,61^ as well as droplet-based microfluidics.^62,63^

In this work, we demonstrate programmable 2D motions of liquid droplets on liquid-infused surfaces (LIS) and within solid-wall microchannels without engineering the substrate. This is achieved by synthesizing two spiropyran/merocyanine-based photoswitches, characterized by their fast kinetics and large polarity changes. These properties enable fast changes in the interfacial tension upon irradiation. The movement on surfaces is achieved by two different mechanisms, namely photo-Marangoni flow for droplet movement on LIS and unbalanced surface tension for liquid movement inside solid channels. We also demonstrate complex 2D movement of droplets on a liquid surface, with migration velocities 2.5× faster than prior work on surface-propagating drops,^64^ and comparable to velocities previously only observed for fully submerged drops.^65^ The surfactants used can be activated by light whose intensity is 1–2 orders of magnitude lower than the laser intensities used in thermal-capillary actuation. We then characterize the magnitude and rate of change in interfacial tensions of various systems, which indicates that the fast-switching kinetics are beneficial for a rapid response to change in interfacial tension and maintain the interfacial tension gradient within a droplet. Furthermore, we present a scaling model to explain the observed droplet-size-dependent velocity, and to predict how migration velocity depends on surfactant properties. The internal flow topology is also visualized using tracer particles; whereas tracer particles have previously been employed to visualize Marangoni flow,^64,65^ we present a direct comparison of their motion with the outcomes from numerical simulations, finding reasonable agreement. The results and techniques demonstrated in this work enable advances for the use of photoresponsive surfactants for dynamic manipulation of multiphase fluid systems for energy, building, thermal management, and microfluidics applications.

## Results

### Working Principle, Synthesis and Characterization of Photosurfactants

**Figure 1:**
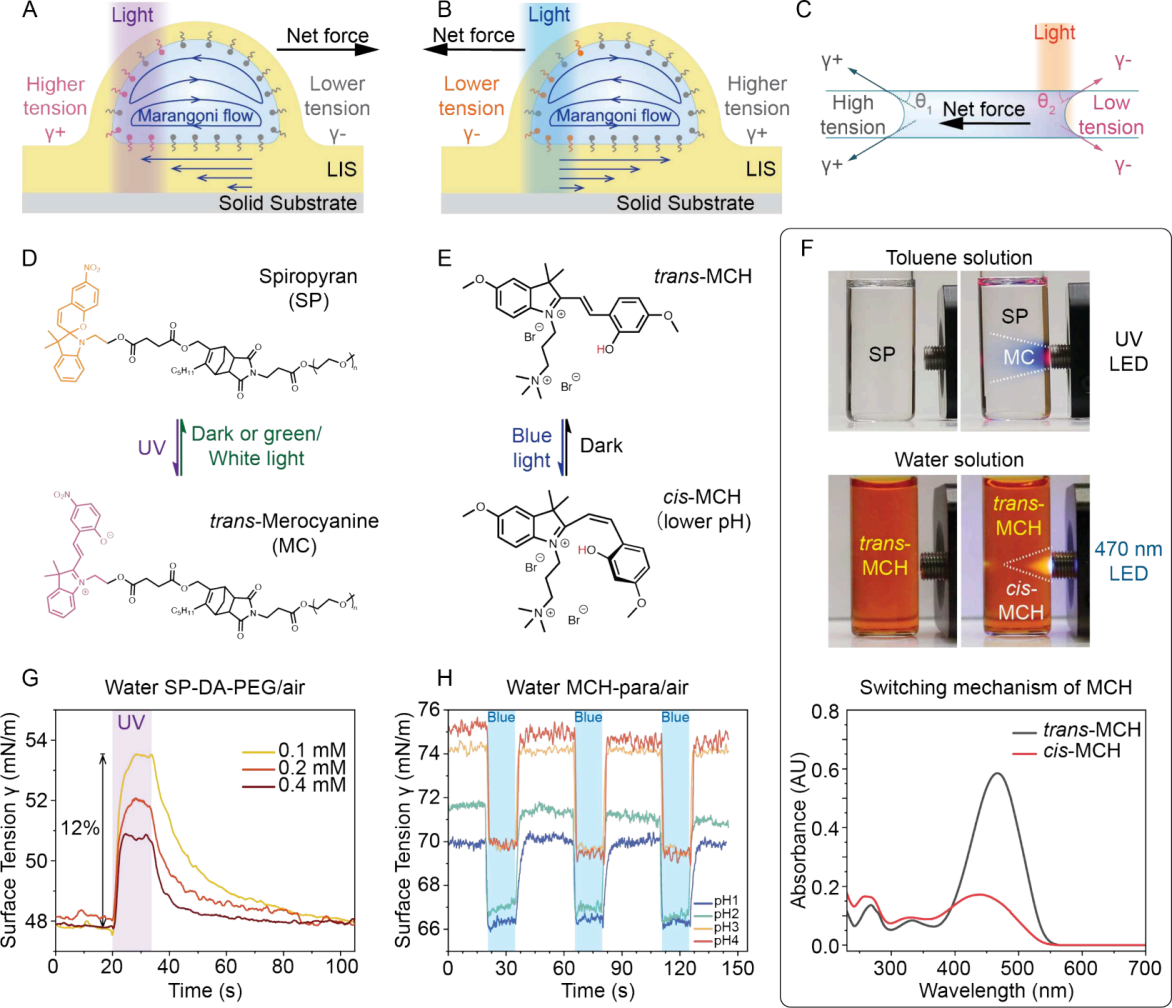
Mechanisms of liquid movement driven by photoresponsive surfactants, molecular structures of the photoresponsive surfactants, and surface tension response. (A) Schematic of a lubricant-cloaked droplet on a liquid-infused surface (LIS). The interfacial tension increases under illumination, causing a Marangoni flow from the unilluminated region to the illuminated region, and a net shear force away from the light. (B) Schematic of a lubricant-cloaked droplet on LIS. The interfacial tension decreases under illumination, causing a Marangoni flow from the illuminated region to the unilluminated region, and a net shear force toward the light. (C) Schematic of liquid in a microchannel or capillary tube. The surface tension changes under illumination, which results in an unbalanced total surface tension force on the liquid column and the subsequent liquid movement. Molecular structures and photoswitching of (D) SP-DA-PEG and (E) MCH-para. (F) The change of color of SP-DA-PEG in toluene under UV illumination and MCH-para in aqueous solution (phosphate buffer, pH 3) under 470 nm illumination. UV–vis spectrum demonstrating the switching mechanism of MCH-para in aqueous solution (pH 1).(G) Surface tension response of SP-DA-PEG in water under UV (365 nm) illumination with an optical intensity of 37.1 mW/cm^2^. (H) Surface tension response of MCH-para in water at various pH levels under blue light (470 nm) illumination with an optical intensity of 31.8 mW/cm^2^.

The working principle for driving fluid motion can be explained by two different mechanisms: interfacial shear caused by a photo-Marangoni (or chromo-capillary) flow and unbalanced surface tension forces. As illustrated in Figure 1A and 1B, for a droplet on LIS consisting of a porous hydrophobic surface infused with lubricant oil, applying light on one side of the droplet will cause the photoresponsive surfactants or molecules under illumination to switch to the metastable isomer. This causes the local surface tension or interfacial tension γ to increase (Figure 1A) or decrease (Figure 1B). Meanwhile, the surfactants on the nonilluminated side remain in the thermodynamically stable form. For droplets cloaked by the lubricant, an interfacial tension gradient will be established, generating a Marangoni flow from the low interfacial tension side to the high interfacial tension side. The interfacial flow generates a net shear force on the droplet, which causes it to move in the direction opposite to the interfacial flow direction. By adjusting the concentration of the surfactants, pH of the solution, and light intensity, the gradient in γ can be quantitatively adjusted. However, for the liquid inside microfluidic channels with solid walls (Figure 1C), no Marangoni flow is present since there is no fluid–fluid interface. Rather, light causes the surface tension on the illuminated side of the liquid column to increase or decrease. The unbalanced surface tension force acting on the liquid along the channel direction becomes the main driving force.

To investigate photoresponsive surfactants with the ability to program fast 2D motion of liquid droplets upon irradiation, we prepared two types of spiropyan/merocyanine based photosurfactants. The first surfactant is Spiropyran-PEG (SP-DA-PEG) bearing an electron-withdrawing nitro group (Figure 1D), which has been previously reported in our work.^46^ The design of SP-DA-PEG includes a photoswitchable spiropyran unit at the tail and a poly(ethylene glycol) monomethyl ether chain as the hydrophilic headgroup. This molecule can be converted between neutral spiropyran (SP) and charged merocyanine (MC) forms upon 365 nm light irradiation (Figure S2); the reverse reaction occurs thermally in the dark when the UV source is removed and can be accelerated by applying green light (∼532 nm). Additionally, a new merocyanine photoacid with two electron-donating methoxy groups incorporated at the *para-* positions in the indolium and the phenolic moiety was synthesized (MCH-para, Figure 1E). Recently, Beves et al. described a detailed switching mechanism of merocyanine photoacids.^66^ The kinetics and the photoswitching properties depend on the equilibrium between the MCH photoreactive states, solvent choice, pH of an aqueous solution, and substituent pattern.^67,68^ At low pHs, the MCH-para switches almost instantaneously between *trans-*MCH and the more acidic *cis-*MCH form upon application and removal of blue light (∼470 nm), respectively (Figure 1E). Such fast-switching kinetics is beneficial to achieve a fast response of surface tension change, and thereby to maintain the surface tension gradient within a migrating droplet. Figure 1F shows that a 1 mM MCH-para aqueous solution bleaches from dark orange to yellow upon irradiation of 470 nm light, using a low-power fiber-coupled blue LED (20 mW, Thorlabs M470F4). The UV–vis spectrum depicting the switching mechanism of MCH-para in an aqueous solution at pH 1 is also shown in Figure 1F. Moreover, we demonstrate that MCH-para has strong hydrolytic stability at low pHs compared to previously reported merocyanine photoacids, which tend to hydrolyze due to the nucleophilic attack of water (Figure S8A).^69^ MCH-para was stable at pH 1–3 at room temperature for more than one month in aqueous media (Figure S8B). This can be attributed to *para-* substitution with electron-donating methoxy groups both in indolium and phenolic moiety.^70^ Both molecular photoswitches, SP-DA-PEG and MCH-para, exhibit large polarity changes and return back to their original state within seconds in the dark. The fast-switching kinetics, in both directions, is critical to the surface tension change, and to maintain a stationary interfacial tension gradient within the droplet. This is in contrast to previously reported AzoTAB systems (Table S1), which are also used for fluid manipulation. The thermal half-life reported for these compounds is on the order of 24 h in the dark at room temperature. Because the thermal half-life controls the duration of the light-induced property change, a second light source of different wavelengths or extended time in the dark is needed to regenerate the initial state.^56,71^ Moreover, as noted by Baigl, if the AzoTAB is placed inside the droplet, the recirculation flow rapidly reaches equilibrium inside the droplet due to the long half-life and it is impossible to maintain a stationary interfacial tension gradient.^56^

We measured the surface tension responses of SP-DA-PEG and MCH-para aqueous solutions using a standard pendant drop method on a commercial tensiometer (Theta Flex, Biolin Scientific). Under light illumination, the surface tension of SP-DA-PEG increases (Figure 1G and Figure S14B) while the surface tension of MCH-para solution decreases (Figure 1H). Figure 1G shows that the change in surface tension of the SP-DA-PEG aqueous solution can be tuned by varying the surfactant concentration. In this case, a 0.1 mM solution demonstrated a 5.6 mN/m change in surface tension within 8 s under 37.1 mW/cm^2^ illumination. Under the same illumination, increasing the concentrations to 0.2 and 0.4 mM reduced the response time to 7 and 4 s, respectively, but also resulted in smaller surface tension changes (3.9 and 2.9 mN/m, respectively). In addition, the surface tension response can also be tuned by the light intensity (Figure S14B). The change in surface tension of a 0.2 mM SP-DA-PEG aqueous solution increases from 2.1 to 3.9 mN/m when the light intensity increases from 7.7 to 37.1 mW/cm^2^. These optical intensities are approximately 10^2^ times lower compared to those used for thermocapillary actuation.^72,73^ The reverse switching was on the same time scale (seconds) compared to forward switching. Compared to SP-DA-PEG, the surface tension response of MCH-para in water at various pH levels (phosphate buffer solution) is almost instantaneous (1.1 s for the forward reaction and 1.7 s for the reverse reaction), as illustrated in Figure 1H (∼470 nm, 31.8 mW/cm^2^). We demonstrate multiple cycles of surface tension switching under repetitive pulsed blue light within a short period of time. The change in surface tension varies slightly from 3.6 mN/m at pH 1 to 5.7 mN/m at pH 4. The optimal fast kinetics and enhanced hydrolytic stability of the new photosurfactants are beneficial in a rapid response to interfacial tension changes and maintaining an interfacial tension gradient within a droplet, allowing for sustained motion over long distances and durations.

### Droplet Movement on LIS

**Figure 2:**
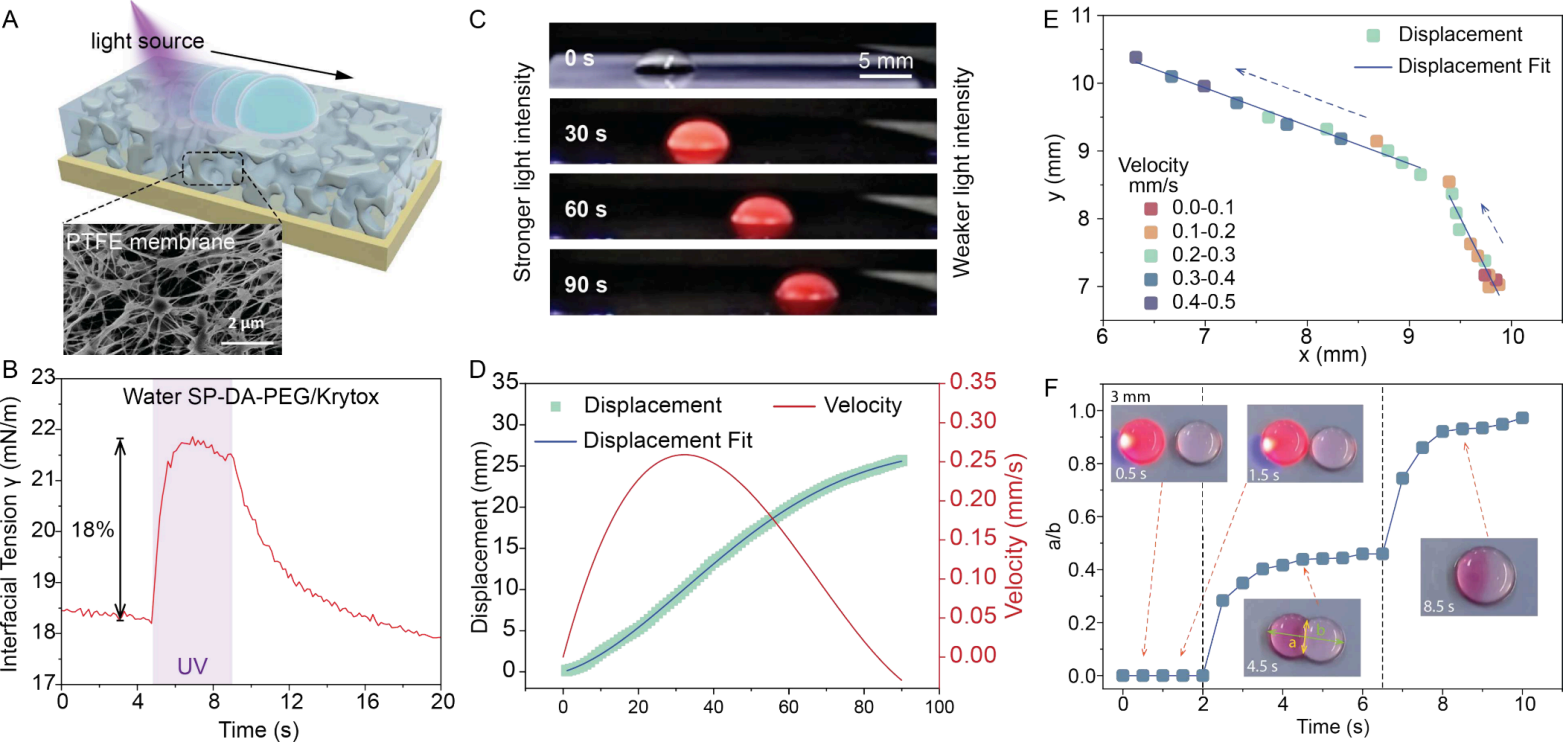
Droplet movement (linear and 2D) and merging on liquid-infused surfaces (LIS) using SP-DA-PEG. (A) Schematic of a liquid droplet on LIS and scanning electron microscopy (SEM) image of a porous PTFE substrate (B) The interfacial tension response of water-Krytox interface with SP-DA-PEG when illuminated with 365 nm UV at 37.1 mW/cm^2^ optical intensity. (C) Time-lapse optical images (side view) of linear movement of a water droplet containing SP-DA-PEG on LIS directed by UV light with an intensity gradient. Light intensity is stronger on the left side and weaker on the right side. (D) Line plot of the time-dependent displacement and velocity of the droplet in Figure C. (E) Line plot of the displacement and velocity of water droplets containing SP-DA-PEG on LIS changing moving directions on demand, and (F) Top-down view of time-lapse optical images of two droplets merging driven by the photo-Marangoni effect. The line plot depicts a dimensionless distance of the two droplets as a function of time.

We first demonstrate and characterize droplet movement on a liquid-infused surface (LIS) using SP-DA-PEG and MCH-para (Figure 2A). LIS offers the advantages of high droplet mobility and low contact angle hysteresis, which make it particularly suitable as a microfluidic platform for fluid manipulation, as an anti-icing and antifouling surface, and as a condensation surface for power generation, thermal management, and desalination. The LIS used in this study consists of a porous PTFE membrane (Figure 2A) infused with Krytox. As water droplets containing 0.2 mM SP-DA-PEG and 1 mM MCH-para (pH 3) respectively, were deposited on the LIS, we observed interference diffraction fringes on the surface of both droplets (Figure S16A). This suggests that the droplets were cloaked by Krytox. In the absence of Krytox, the surface of the droplet shows no interference patterns (Figure S16B).

Since both SP-DA-PEG and MCH-para are nonsoluble in Krytox, they were anticipated to adsorb to the water-Krytox interface. For SP-DA-PEG, the water-Krytox interfacial tension was measured to increase from 18.2 to 21.8 mN/m (∼20% increase) after UV illumination (365 nm, 110 mW/cm^2^), as shown in Figure 2B. The increase in interfacial tension is consistent with the increase of surface tension of water under UV (Figure 1G). Therefore, the application of UV light to one side of the droplet containing SP-DA-PEG will increase the interfacial tension locally. This generates a Marangoni flow from the unilluminated side to the illuminated side on both the top and bottom interfaces of the droplet, and two vortices form inside the droplet. A velocity gradient develops in the Krytox in LIS as a result of the interfacial flow, which exerts a net shear force on the droplet in the direction away from the light (Figure 1A).

The mechanism described above for SP-DA-PEG is confirmed in Figure 2C and Movie S1. When a water droplet containing SP-DA-PEG is placed in an environment where the light intensity is stronger on the left side and weaker on the right side realized using a continuously variable neutral density filter (Thorlabs NDL-10C-2) and a UV lamp (Analytik Jena, UVP BLAK-RAY B-100AP ALMP), the droplet moves toward the darker region (i.e., away from the light). Although the velocity is relatively slow (∼250 μm/s, Figure 2D red solid line), this is the first demonstration of droplet movement on LIS activated by photoresponsive surfactants. The output optical intensity after the UV light passed through the center of the filter was very low (6.1 mW/cm^2^). In comparison, an equal-sized water droplet containing no surfactants remained stationary under the same illumination conditions (Figure S18), suggesting that droplet movement was not caused by the thermal-Marangoni effect from UV heating. Furthermore, the moving direction of the droplet can be controlled on the fly.

As shown in Movie S2 and Figure S15, a water droplet was initially moving in the + *y* direction driven by a UV laser (351.1 and 363.8 nm, beam diameter of 1.3 mm, optical power of 101 mW). Upon changing the laser position, the droplet immediately moved in the – *x* direction. The trajectory and velocity of the droplet are depicted in Figure 2E. The photo-Marangoni effect can also achieve controlled merging of multiple droplets. As illustrated in Figure 2F and Movie S3, the same UV laser can drive a droplet to move toward and merge with another droplet. Figure 2F shows a dimensionless distance (a/b) of the two droplets as a function of time, defined in the inset of Figure 2F. The merging process consists of two stages. Initially, the droplets touch without completely combining into a single droplet, possibly due to the intervening cloaking oil (*t* = 2 s to *t* = 6.5 s). Subsequently, after remaining in this metastable state, the droplets fully merge, presumably when the oil film ruptures (*t* > 6.5 s).

**Figure 3:**
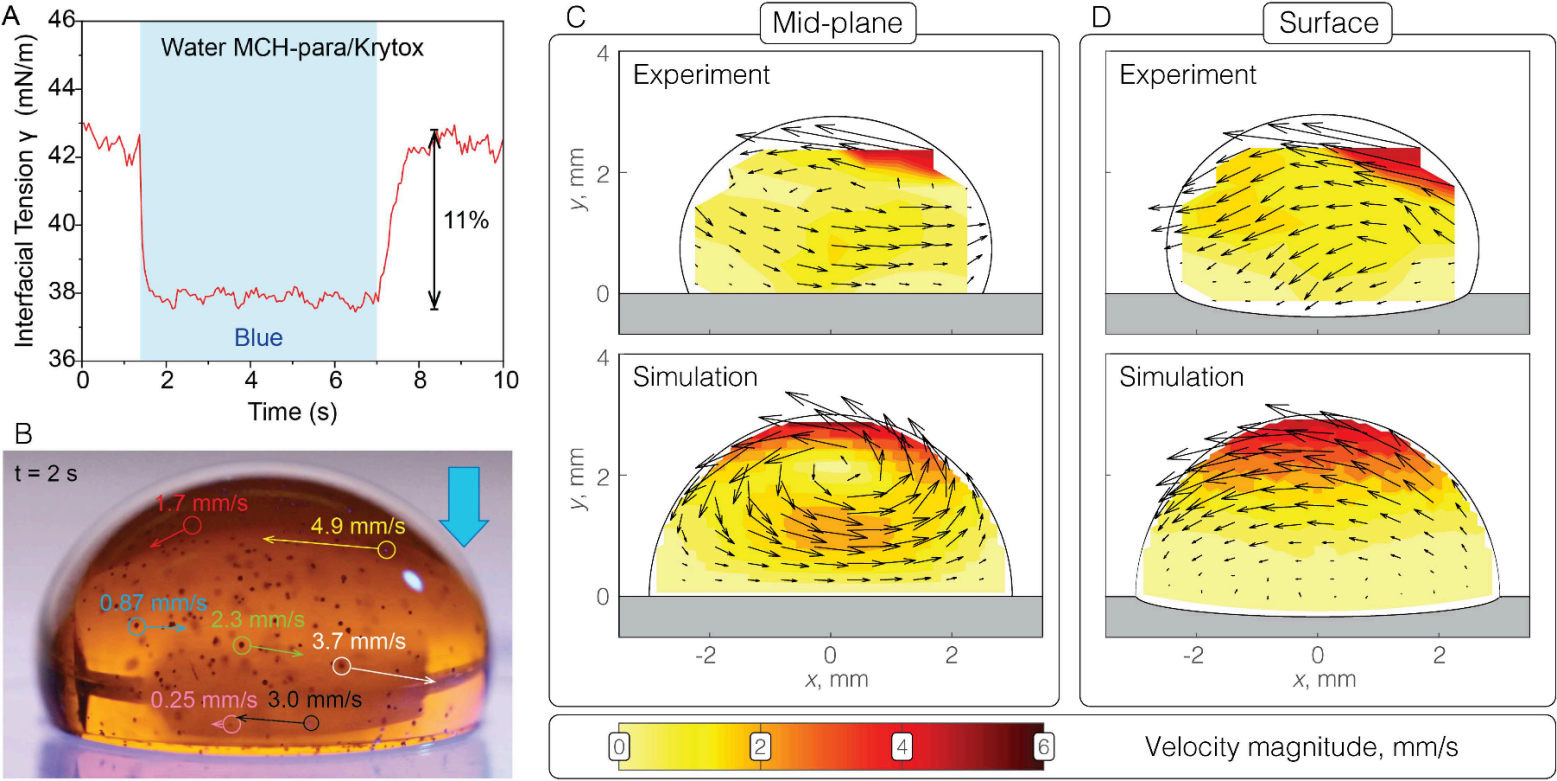
Droplet movement on liquid-infused surfaces (LIS) using MCH-para. (A) The interfacial tension response of water-Krytox interface containing MCH-para when illuminated with 470 nm at 31.8 mW/cm^2^ optical intensity. (B) Images (side view) of the internal flow field indicated by tracer particles of a water droplet containing MCH-para. Illumination at 470 nm was applied on the right side of the droplet. (C,D) Comparison of experimental and computational results for the flow velocities. (C) Results near the midplane of the droplet and (D) close to the surface of the droplet. The orientations and lengths of the arrows represent the directions and magnitudes of the local flow velocity. The blank regions in white in the experiments have no data points, due to reflections near the outline of the droplet.

We further investigated the movement and the internal flow field of water droplets (pH 3) containing 1 mM MCH-para on LIS (Figure S19). In contrast to SP-DA-PEG, the water-Krytox interfacial tension with MCH-para decreased from 42.9 to 37.9 mN/m (Figure 3A, 470 nm, 31.8 mW/cm^2^); the magnitude of this change is also consistent with our surface tension measurement (Figure 1H). This reduction in the interfacial tension is also evident in the contact angle measurement (Figure S17), where we observed a reduction from 117° to 113° upon irradiation. Due to the decrease of interfacial tension with irradiation, we expect that the droplet containing MCH-para will move toward the light, and therefore in the opposite direction compared to SP-DA-PEG drops, as sketched in Figure 1B.

The droplet’s internal flow was examined using tracer particles (Cospheric, sliver-coated hollow glass microsphere 25–65 μm, 0.9 g/cm^3^). Particle tracking velocimetry in ImageJ was used to calculate flow velocities (Figure 3B and Figure S20). To provide approximate flow fields, velocity tracks were processed in MATLAB. Velocities were shifted to (steady) frame of reference translating with the droplet, by subtracting the migration velocity from the horizontal velocity components. Velocities were then mapped to a regular grid using a binning algorithm based on the median of track positions nearest to each grid point. This analysis provided insights on the local velocities at the droplet’s surface and its midplane (Figure 3C and 3D). In Figure 3C, the velocities of particles moving at the midplane reach up to 6 mm/s, corresponding to the magnitudes observed in the video.

To further our understanding, and to assess whether the flow could be reproduced by a computationally manageable simulation, a numerical model was developed using COMSOL Multiphysics. This model was designed to qualitatively represent the internal flow field. In a reference frame moving with the droplet, the model assumes a steady flow; for simplicity, the drop shape is assumed to be a hemisphere. The model assumes that a shear stress is exerted on both the top and bottom interfaces of the water droplet, which is cloaked by a thin layer of lubricant (50 μm). The magnitude of the applied shear is estimated using a scaling model accounting for surfactant advection and photoswitch, as outlined in the next section (and described in detail in the Supporting Information). The scaling model predicts a stress of order 0.1 Pa. We found that a stress of 0.14 Pa provided agreement in terms of flow velocities, migration velocity, as well as flown patterns, as explained below. Care was taken to refine the computational grid at the drop boundaries and inside of the thin oil layers, and weak constraints enforced surface stresses as well as steady migration at an unknown velocity, which was computed as part of the solution. The model had approximately 271,000 degrees of freedom; a converged solution was obtained in approximately 5 min.

The drop migration velocities in the experiment and simulation are 0.11 and 0.10 mm/s, respectively. The directions and velocities of the fluid flow predicted by the model were compared with our experimental observations, as depicted in Figure 3C and 3D. Figure 3C shows experimental and computational data near the drop midplane: the top part of the droplet shows a recirculation pattern, with the maximum velocity occurring at the top. The flow initiates from the side exhibiting lower interfacial tension and proceeds along the surface, as also evidenced in Figure 3D, before recirculating through the central region. By combining our observations of the flow at the droplet’s bottom with the surface and midplane flow data, we inferred that the photo-Marangoni flow traverses both the top and bottom surfaces following the tension gradient and then cycles back through the bulk (interior) fluid. Note that the return flow in the interior is slower than the surface flow. These results also show that the key features of this photosurfactant flow can be reproduced by a computationally inexpensive simulation.

### Droplet Transport on Liquids

**Figure 4:**
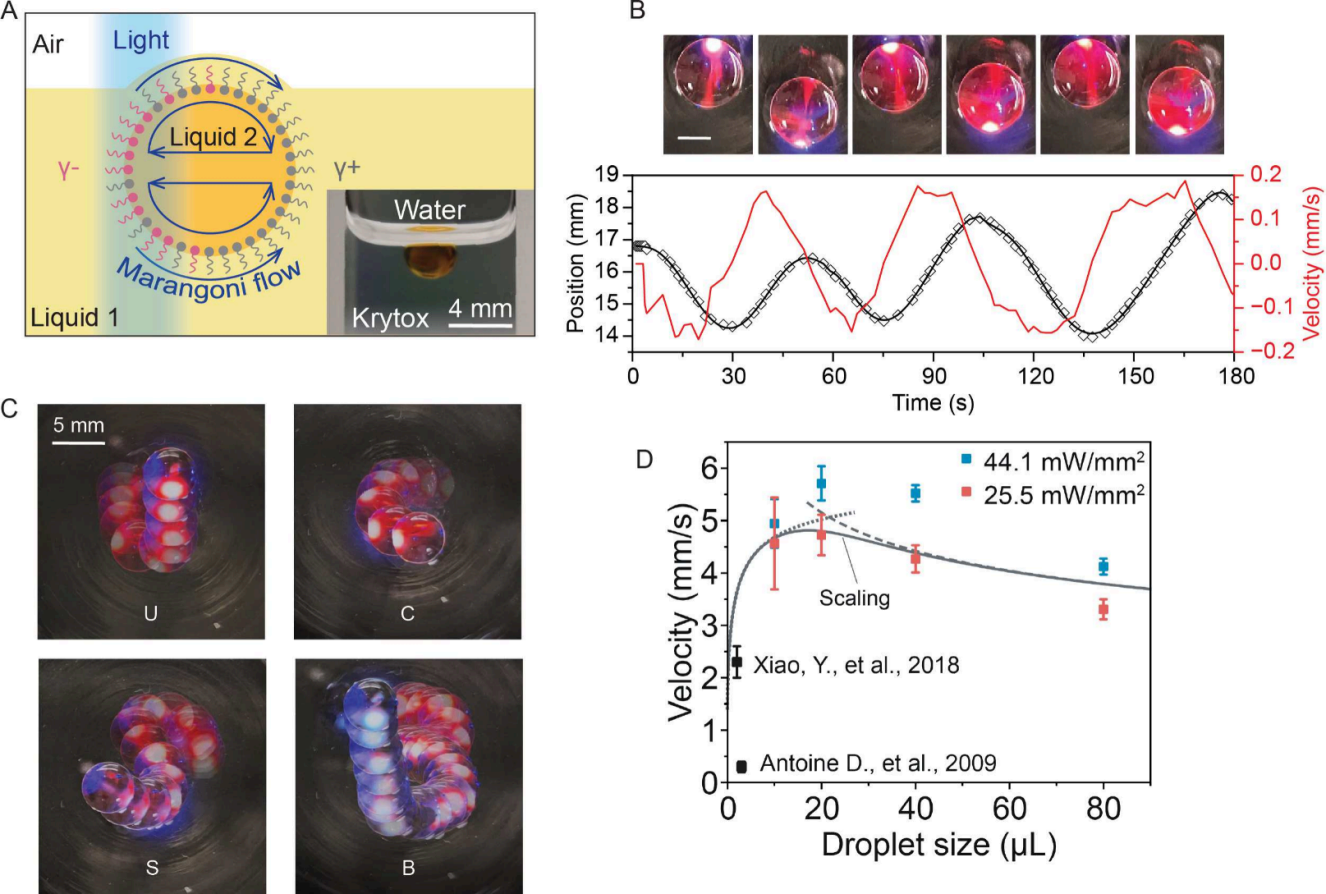
Liquid movement (linear and 2D) on another immiscible liquid and inside solid-wall microchannels. (A) Schematic and optical image of a liquid droplet floating at the surface of an immiscible liquid. (B) Time-lapse optical images (top-down view) of linear movement and switching of the direction of a water droplet containing SP-DA-PEG on Krytox, directed by a UV laser. The corresponding displacement of the droplet (black line) and its velocity (red line) as functions of time are also shown. The scale bar is 3 mm. (C) Overlaid images (top-down view) of SP-DA-PEG droplet trajectories spelling the letters “UCSB”, driven by a UV laser. The time interval between images is 10 s. (D) The average moving velocity of SP-DA-PEG droplets as a function of droplet volume and light intensity under UV illumination. Scalings for small and large drops are shown by dotted and dashed lines; a composite is shown by the continuous line. The results of refs (47,56) are shown for reference.

We further investigate liquid motion directly on another immiscible liquid as a limiting case where there is no porous PTFE in the substrate (Figure 4A). Linear droplet motions on a liquid substrate^47,56^ and immersed in liquid mediums^54,55^ have been previously investigated. In this work, we demonstrate the capability to form complex patterns, and achieved 2.5 times higher maximum droplet velocity. The higher velocity is attributed to the fast kinetics and significant polarity of the newly developed surfactants. Water droplets containing 0.2 mM of SP-DA-PEG were placed on a thick layer (>1 cm) of higher-density Krytox lubricant. As shown in Figure 4B and Movie S5, the application of a UV laser (351.1 and 363.8 nm, beam diameter of 1.3 mm, optical power of 101 mW) can actuate droplet motion and switch its moving direction repeatedly with a maximum velocity approaching 0.2 mm/s. Moreover, we show in Figure 4C and Movie S6 that the same laser can precisely drive droplets to describe a complex pattern, shown here by spelling the letters “UCSB”. Figure 4C comprises overlaid time-lapse images to illustrate the trajectory of the droplet. Consistently with our previous experiments, droplets containing SP-DA-PEG move away from the light, as shown in Figure 4B and 4C, whereas droplets containing MCH-para move toward the light, as indicated in Supporting Information.

Furthermore, we studied how velocity depends on size and light intensity. Figure 4D shows the average moving velocity of SP-DA-PEG droplets as a function of droplet volume and light intensity. The maximum velocity was observed when the droplet volume was approximately 20 μL, which is 2.5 times higher compared to previous studies.^47,56^ Velocity decreases gradually as drop volume is increased past 20 μL.

We suggest simple scaling arguments to explain the observed maximum in drop velocity. Within the drop, interfacial tension increases rapidly for fluid within the laser spot; as the fluid moves away from the light, its interfacial tension gradually returns toward its “dark” value (as shown earlier in Figure 2B). For smaller volumes, fluid takes less time to travel the drop length, leading to an incomplete reverse photoreaction and therefore a smaller decrease in interfacial tension. As a result, the interfacial tension difference across the drop becomes weaker. Balancing this Marangoni force and the fluid resistance (see Supporting Information), we obtain the dotted curve for the velocity *V* in Figure 4D. For small drops
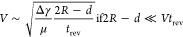
1where μ is the viscosity of Krytox, *R* the drop radius, *d* the beam diameter, *t*_rev_ the reverse reaction time scale, and Δ γ the maximum surface tension change achievable under the given illumination (see Supporting Information). For larger drops, there are several contributing factors that could explain the decrease in velocity with increasing volume. We note for example that the largest drops have Reynolds numbers , such that some of the Marangoni force is expended to overcome the inertia of the surrounding fluid, thereby decreasing propulsive efficiency. A scaling based on this hypothesis is shown by the dashed line in Figure 4D, which approximately follows *V* ∼ *R*^–1/2^ (see Supporting Information); the continuous line is a composite of the results for small and large drops.

Previous studies reported that the photo-Marangoni effect occurs in isothermal conditions (i.e., without localized heating). However, we note that heating from light absorption is possible.^74^ Here, we show that under the experimental conditions investigated in our study, the photo-Marangoni effect is the dominant factor despite the coexistence of a thermal-Marangoni effect. For SP-DA-PEG, illumination causes the surface tension and interfacial tension to increase, while increased temperature causes a decrease. By inserting a thermocouple inside a droplet, we measured that UV LED caused a less than 2 °C temperature rise and the UV laser caused a less than 6 °C temperature rise (Figure S12). In all of our experiments, the samples were positioned on an optical table which effectively dissipates heat. The direction of fluid motion further corroborates that the stress from the photo-Marangoni effect largely suppresses the influence of temperature variations. However, for MCH-para solutions, since both heating and photoswitching decrease the surface and interfacial tension, we measured the change of surface tension caused purely by an increase in temperature from 20 to 60 °C using a custom syringe heating system (Figure S9A). Surface tension decreases with temperature following the equation
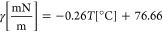
2To estimate the separate contributions from heating and from photoswitching on the change of surface tension shown in Figure 1H, we measured the temperature rise of a water droplet (pH 3) containing MCH-para under the same illumination condition (470 nm 31.8 mW/cm^2^) using an infrared camera (Telops, M3K, Figure S10A). We used the emissivity of pure water (ϵ = 1) to approximate the emissivity of the MCH-para aqueous solution (Figure S9C). As shown in Figure S10C and S10D, the highest temperature within the MCH-para droplet increased from 19.6 to 20.6 °C after 150 s of blue light illumination (31.8 mW/cm^2^). According to eq 2, this temperature change only generates a 0.26 mN/m decrease in surface tension, which is only 5.9% of the surface tension change in Figure 1H. Furthermore, the temperature increase is negligible within the time scale of the photoswitch (<2 s). Therefore, under these optical intensities, even though both heating and photoswitching contribute to a decrease in the surface tension, the photoisomerization-induced Marangoni effect is the primary factor in driving droplet motion. When applying a higher optical intensity, the surface tension changes caused by heating and photoswitching both increase in their magnitude; their relative contributions can be estimated by the method presented here.

### Liquid Transport in Microchannels

**Figure 5:**
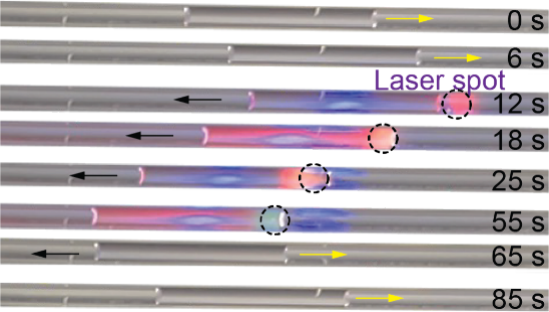
Time-lapse optical images (side view) of a toluene liquid column containing SP-DA-PEG moving inside a glass capillary tube directed by a UV laser.

In addition to modulating droplet movement on LIS and liquid surfaces, here we also demonstrate the direct movement of liquid on solid walls of microchannels or capillary tubes using light-responsive surfactants. Figure 5 shows time-lapse side view images of a glass capillary tube (0.5 mm diameter) containing toluene with 0.1 mM SP-DA-PEG surfactants (Movie S7). The glass tube was intentionally tilted so that under the influence of gravity, the liquid naturally slide toward the right (from 0 to 6 s). Application of light (351.1 and 363.8 nm, beam diameter of 1.3 mm, optical power of 101 mW) on the right side of the liquid column caused the liquid to move toward the left (12 to 55 s). When the light was removed, the liquid resumed moving toward the right due to gravity (65 to 85 s). In our previous work, the surface tension of toluene containing SP-DA-PEG was measured to decrease with UV illumination.^46^ Since the only fluid–fluid interface is the toluene-air interface at the two ends of the liquid column, the mechanism driving fluid motion is not the Marangoni effect, but rather, the unbalanced surface tension forces on the liquid. Specifically, the surface tension on the left unilluminated side remains the same whereas the surface tension on the right illuminated side reduces, contributing to a net force driving the liquid to move away toward the higher surface tension side. In addition, a change in contact angle can also contribute to the horizontal component of the net surface tension force (Figure 1C). However, we did not observe a clear change in the contact angle with and without illumination.

## Discussion

We demonstrated a novel approach for dynamic manipulation of droplet motions on lubricant-infused surfaces (LIS), solid-wall microchannels, and on liquid substrates using photoresponsive surfactants designed and synthesized by us. The surfactants MCH-para and SP-DA-PEG selected in this study have different reaction time scales (1 to 10 s) and cause surface tension and interfacial tension to change in the opposite direction. MCH-para is particularly stable in low-pH aqueous solutions. In addition, the photoswitch is activated with UV or blue light with optical intensity 1–2 orders of magnitude lower than what is typically used by thermal-capillary actuation. We show for the first time that droplets are able to move and merge in linear motions on LIS, and their moving directions can be changed dynamically by light. The Marangoni-induced internal fluid flow was also visualized by analyzing the trajectory and velocity of tracer particles, which confirmed our proposed mechanism. In particular, the moving direction depends on if surfactants increase or decrease the surface or interfacial tension. Furthermore, we show fast motion (up to 5.8 mm) of droplets floating on another liquid substrate, and that droplets are able to move in arbitrary directions on a 2D surface to form complex patterns. We developed scaling arguments to explain the observed droplet-size dependent velocity and a simplified numerical model to qualitatively illustrate the internal flow.

In addition to the photo-Marangoni effect, we also demonstrate the movement of liquid inside solid-wall microchannels which is caused by unbalanced surface tension along the moving direction. This can potentially be used as a noncontact pumping mechanism for microfluidic applications.

The droplet manipulation platform presented in this work opens doors for applications requiring noncontact, noninvasive, dynamically tunable and programmable fluid motions with minimal external energy input. With the use of light and photoresponsive surfactants, our approach does not require any micro/nanofabrication. Light can also be focused down to the diffraction-limit length, potentially enabling microfluidic manipulation down to the 1-μm spatial resolution. The surfactant incorporation may influence the general applicability of the droplet manipulation. For example, they need to dissolve in one of the involved liquid phases, and in biological applications, the surfactants must be biocompatible. By incorporating the functional groups into the molecules, the photoresponsive surfactants show substantial potential for broader applications. This approach requires surfactants to be designed with significant polarity changes to induce surface tension changes and fast switching kinetics when applying and removing light, thus creating the gradient change for droplet manipulation. By choosing the appropriate solvent, we have demonstrated that the surface tension varies in solvents with different polarities. In this work, SP-DA-PEG is soluble in water and toluene, while the MCH-para molecule is specifically designed for aqueous solution applications for safety purposes. Moreover, photosurfactants that can be soluble in the lubricant of LIS can help with droplet removal in condensation processes, which can potentially enhance condensation heat transfer significantly for power generation and desalination applications. The approach presented here also serves as a powerful method for multiphase processes in microgravity, where droplet motions cannot be achieved with gravity.

## Materials and Methods

### Sample Preparations

The photoactive surfactant SP-DA-PEG used in this study is soluble in toluene and water. The solutions with the desired concentration were prepared by sonicating appropriate amount of SP-DA-PEG in solvents (35). The photoactive surfactant MCH-para used in this study has a high solubility in aqueous solutions. A concentrated stock solution was used to create a 1 mM MCH-para solution. The stock solution of MCH-para (C = 2 mM) was prepared by adding 15 mg (Molecular Weight = 584.38 g/mol, 0.026 mmol) in 12.83 mL of deionized water in a 20 mL vial, wrapped in aluminum foil and stored at 4 °C. Final solutions were prepared in a 4 mL cuvette by adding 0.6 mL of DI water followed by 0.4 mL of corresponding buffer solution (pH 1–4) and equilibrated for 10 min. After which, 1 mL of MCH-para stock solution was added and equilibrated for 15 min in the dark.

### Materials

Surfactants, SP-DA-PEG and MCH-para, were synthesized in the laboratory (SI Appendix, Note S1). Buffer solutions were prepared in the laboratory by adding NaOH or HCl to Na_2_HPO_4_ and NaH_2_PO_4_ solutions (reagents were obtained from Sigma-Aldrich, Oakwood Chemical, or Fisher Scientific). Solvents, deionized water and toluene, were purchased from Sigma-Aldrich. Lubricant oil Krytox General-Purpose Oils (GPL) 100, was supplied by Miller-Stephenson. This oil is composed of a perfluoropolyether (PFPE) base, also known as perfluoroalkylether (PFAE) or perfluoropolyalkylether (PFPAE). Its molecular structure is fully saturated, comprising carbon, oxygen, and fluorine atoms.^75^ The carbon–fluorine bond in Krytox GPL 100 is characterized by high binding energy, rendering it exceptionally stable both chemically and thermally.^76^ As a result, Krytox lubricants are notably chemically inert. This chemical structure also contributes to the significant hydrophobic properties of the oil. PTFE thin films were obtained from Sterlitech (PTFE unlaminated membrane filters, 0.2 μm, 90 mm; the typical porosity of the PTFE is 90%). SiO_2_ microsepheric tracer particles were obtained from Cospheric (sliver-coated hollow glass microsphere 25–65 μm, 0.9 g/cm^3^). All reagents were of analytical grade and used as received.

### Light Sources

A custom multiline argon ion UV laser (351.1 and 363.8 nm) with a beam diameter of 1.3 mm and maximum light intensity of 44 mW/mm^2^ was used for SP-DA-PEG droplet movement experiments. The fiber-coupled 365 nm UV LED (M365FP1, 9.8 mW Min Fiber-Coupled LED), 470 nm blue LEDs (M470F3, 17.2 mW Min Fiber-Coupled LED and M470L3-C1, 350 mW Collimated LED), 530 nm green LED (M530F2, 6.8 mW Min Fiber-Coupled LED) and white LED (MCWHF2, 21.5 mW Min Fiber-Coupled LED) were directly bought from Thorlabs, Inc. The blue laser was built with complete laser diode (LD) operation starter sets from Thorlabs, Inc., including a laser diode controller (LDC 200C), a thermoelectric temperature controller (TED 200C), and a 450 nm blue laser diode (L450P1600MM 1600 mW, 5.6 mm, G Pin Code, MM). A high-intensity UV lamp (216 mm × 140 mm, UVP High Intensity Lamp, Analytik Jena) coupled with an ND filter (Thorlabs NDL-10C-2) was used to conduct experiments on driving SP-DA-PEG droplet motion.

### Instrument and Characterization

Surface tension, interfacial tension and contact angle were characterized using a standard pendant drop method on commercial tensiometer, Biolin Scientific, Theta Flex. Optical power was measured by using optical power meter (Newport, 843-R) connected with a thermopile Sensor (Newport 919P-003–10, 3 W, 10 mm, 0.19–11 μm). Optical images and videos were taken by a Canon EOS 80D camera. IR images were recorded by a high-performance Telops M3K infrared camera. UV–Vis absorption spectra were recorded on an Agilent 8453 UV–Vis spectrometer from 200 to 1200 nm wavelengths. ^1^H and ^13^C NMR spectra were recorded on a Bruker 500 MHz NMR spectrometer. The pH of the solutions was measured by using a Thermo Scientific Orion StarA111 Benchtop pH Meter. The scanning electron microscope (SEM) image was obtained from a FEI Sirion SEM.

### Fabrication of the Lubricant-Infused Surface (LIS)

A porous thin film PTFE was obtained from Sterlitech. PTFE film is cut into pieces and adhered to a flat surface, such as a silicon wafer or a microscopy slide. Extra ethanol was applied to the PTFE and allowed to completely infiltrate before evaporating at room temperature in the fume hood. To create the lubricant-infused surface (LIS), inject Krytox GPL 100 lubricant oil into the PTFE that was adhered to the solid, flat surface.

### Safety Statement

No unexpected or unusually high safety hazards were encountered.

## Data Availability

The data described in this article are available in figshare at 10.6084/m9.figshare.22732736 and 10.6084/m9.figshare.21866181.

## Supporting Information Available

Movie S1: Linear movement of a water droplet containing SP-DA-PEG on LIS (MP4)
Movie S2: Water droplets containing SP-DA-PEG changing moving directions on LIS (MP4)
Movie S3: Water droplets containing SP-DA-PEG merging on LIS (MP4)
Movie S4: Internal flow field indicated by tracer particles of a water droplet containing MCH-para (MP4)
Movie S5: Linear movement and switching of the direction of a water droplet containing SP-DA-PEG on Krytox (MP4)
Movie S6: Droplet trajectory depicting a “UCSB” pattern. (MP4)
Movie S7: Toluene liquid column containing SP-DA-PEG moving inside a glass capillary tube (MP4)
Supporting information text, including synthesis and characterization, materials and instrumentation, and other types of characterization; supplemental results, including ^1^H NMR, UV–vis spectra, and additional data as mentioned in the text (PDF)
