# Supplementary material for: Dynamic Manipulation of Droplets on Liquid-Infused Surfaces Using Photoresponsive Surfactant
Source: ACS Cent Sci. 2024 Feb 27;10(3):684–94. doi: 10.1021/acscentsci.3c00982 (PMC10979485; doi:10.1021/acscentsci.3c00982)
Supplement: Supplementary file 8 — oc3c00982_si_008.pdf [file oc3c00982_si_008.pdf]

Supporting Information for

Dynamic Manipulation of Droplets on Liquid-Infused Surfaces using Photo-Responsive Surfactant

Xichen Liang[a,1], Kseniia M. Karnaukh[b,1], Lei Zhao[c], Serena Seshadri[b], Austin J. DuBose[b], Sophia J. Bailey[b], Qixuan Cao[d], Marielle Cooper[c], Hao Xu[c], Michael Haggmark[a], Matthew E. Helgeson[a], Michael Gordon[a], Paolo Luzzatto-Fegiz[c,2], Javier Read de Alaniz[b,2], and Yangying Zhu[c,2]

[a]Department of Chemical Engineering, University of California at Santa Barbara, Santa Barbara, California 93106-5070, USA
[b]Department of Chemistry, University of California at Santa Barbara, Santa Barbara, California 93106-5070, USA
[c]Department of Mechanical Engineering, University of California at Santa Barbara, Santa Barbara, California 93106-5070, USA
[d]Department of Physics, University of California at Santa Barbara, Santa Barbara, California 93106-5070, USA
[1]X.L.and K.K. contributed equally to this work.
[2]To whom correspondence should be addressed. E-mail: pfegiz@ucsb.edu, jalaniz@ucsb.edu, yangying@ucsb.edu

February 26, 2024

This PDF file includes:

Supporting text
Figure S1 to S21
Table S1
Legends for Movies S1 to S7
SI References

Other supporting materials for this manuscript include the following:

Movies S1 to S7

Supporting Information Text

Synthesis and Characterization of SP-DA-PEG and MCH-para

Materials and Instrumentation

All reagents were obtained from Sigma Aldrich, Oakwood Chemical, or Fisher Scientific and were used as received without further purification unless specified. Acetonitrile and dichloromethane were dispensed from a solvent purification system immediately before use or stored over 3Å molecular sieves. Ethanol and chloroform were stored over 3Å molecular sieves before use. Analytical thin-layer chromatography (TLC) was performed with Merck silica gel 60 F254 pre-coated glass plates and visualized by UV light or stained with p-anisaldehyde. ^{1}H and ^{13}C NMR spectra were recorded on a Bruker 500 MHz NMR spectrometer. Chemical shifts are reported relative to residual solvent peaks (δ 7.26 ppm for $CDCl_3$, 2.50 ppm for DMSO-d_6:) in ^{1}H NMR, and δ 77.20 ppm for $CDCl_3$, 39.52 ppm for DMSO-d_6 in ^{13}C NMR).

UV–Vis spectroscopy and UV–Vis kinetic measurements

UV–Vis absorption spectra were recorded on an Agilent 8453 UV–Vis spectrometer from 200 to 1200 nm wavelengths. The photoinduced optical absorption kinetics of SP-DA-PEG and MCH-para were measured on a custom pump-probe setup. The pump beam was generated by a high-power LED (light-emitting diode) source (Thorlabs) coupled into a multimode optical fiber terminated with an output collimator. The UV pump source was generated by the fiber-coupled UV LED (Thorlabs M365FP1, 365 nm, 9.8 mW Min Fiber-Coupled LED); the green pump source was produced by green LED (Thorlabs M530F2, 530 nm 6.8 mW Min Fiber-Coupled LED) and the blue pump source by blue LED (M470F3, 17.2 mW Min Fiber-Coupled LED), which was positioned to illuminate the sample directly. The probe beam was generated by a High-Power MINI Deuterium Tungsten Halogen Source (Ocean Optics DH-MINI) w/shutter 200-2000 nm coupled into a multimode optical fiber terminated with an output collimator. The probe light was altered by a shutter (Uniblitz CS25) and controlled manually or through a digital output port (National Instruments USB-6009) using the LabVIEW program. A 10x10 mm^2 rectangular spectrophotometer cell was placed in a sample holder. The solution was continuously stirred by a miniature stirring plate inserted into the sample holder (Starna Cells SCS 1.11). A system of lenses directed the probe beam into the detector (Ocean Optics Flame-S1-XR spectrometer), which acquired the spectra of the probe light. The detector was connected to a PC via a USB port. The experiment was controlled by a National Instrument LabVIEW program which collected the probe light spectra, determined sample optical absorption spectra, and controlled pump and probe light sources. Kinetics experiments with SP-DA-PEG were performed in 0.05 mM solutions (SI Appendix, Figure S1A-B).

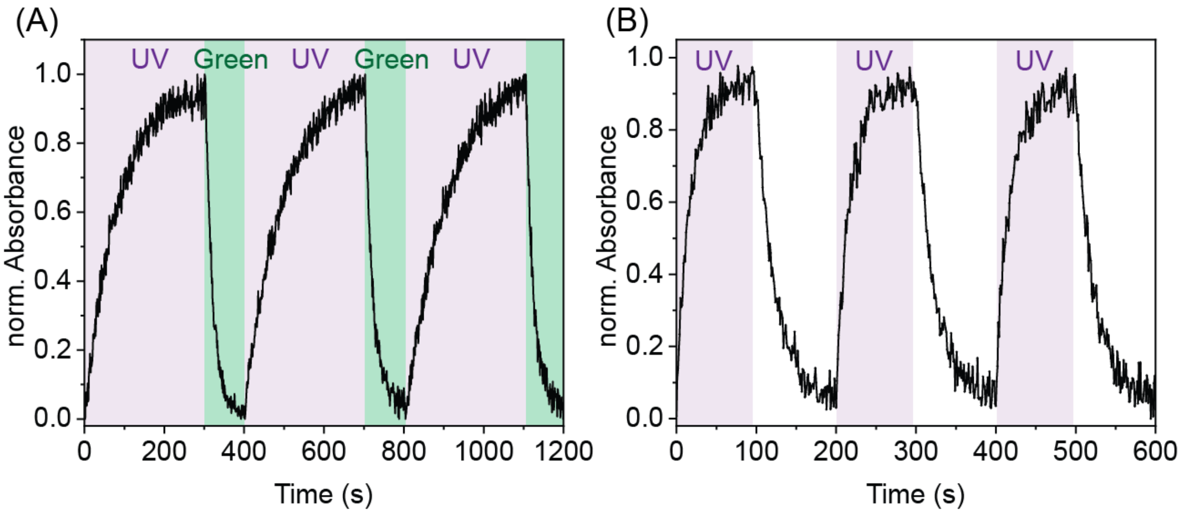

Figure S1: Pump-probe kinetics measurements of SP-DA-PEG in (A) water with alternative 365 nm and 530 nm lights and (B) toluene irradiated with 365 nm light.

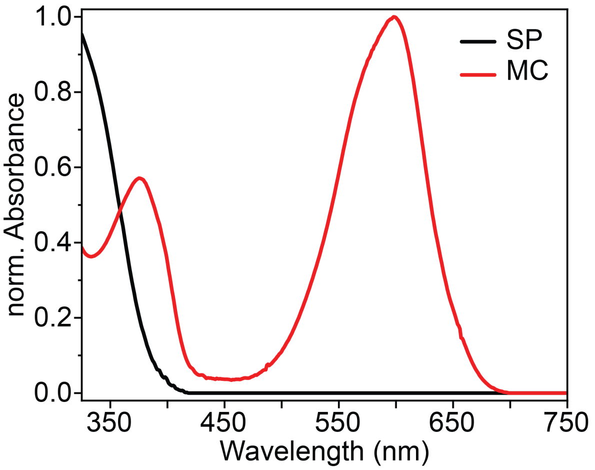

Figure S2: Switching mechanism of SP-DA-PEG in toluene solution.

Solution preparation for the experiments and UV-Vis studies

Solution preparation procedures were modified from Wimberger et al. (*1*).

1. Monobasic buffer preparation: Buffer solutions ranging from pH 1-10 were prepared by adding NaOH (1 M) or HCl (1 M) to Na_2HPO_4 and NaH_2PO_4 solutions (100 mM). DI water was used for all solutions and buffers. The pH of the final solutions was measured by Thermo Scientific Orion Star™ A111 Benchtop pH Meter. Prepared buffers were used for both UV-Vis studies and experiments.

2. The following procedure was used for experiments: The stock solution of MCH-para (C = 2 mM) was prepared by adding 15 mg (Molecular Weight = 584.38 g/mol, 0.026 mmol) in 12.83 mL of DI water in 20 mL vial, wrapped in aluminum foil and stored at 4 °C. Final solutions were prepared in a 2 mL cuvette by adding 0.6 mL of DI water followed by 0.4 mL of corresponding buffer solution (pH 1-4) and equilibrated for 10 minutes. After which, 1 mL of MCH-para stock solution was added and equilibrated for 15 minutes in the dark.

3. UV-Vis samples were prepared as follows: The stock solution of the MCH-para (C = 0.25 mM) was prepared by adding 14.6 mg (Molecular Weight = 584.38 g/mol, 0.025 mmol) in 100 mL of DI water into a volumetric flask wrapped in aluminum foil and stored at 4 °C. Solutions for UV-Vis analysis were prepared in a 1 cm cuvette by adding 0.57 mL of the corresponding phosphate buffer (pH 1-10) followed by 2.052 mL of DI water, placed in the spectrophotometer, and allowed to equilibrate thermally for 10 minutes. Then 0.228 mL of MCH-para stock solution was added to the final concentration of C = 0.020 mM, with continuous stirring of the solution. Scans were collected after 15 minutes of equilibration in the dark.

Synthesis

1. Synthesis of SP-DA-PEG:

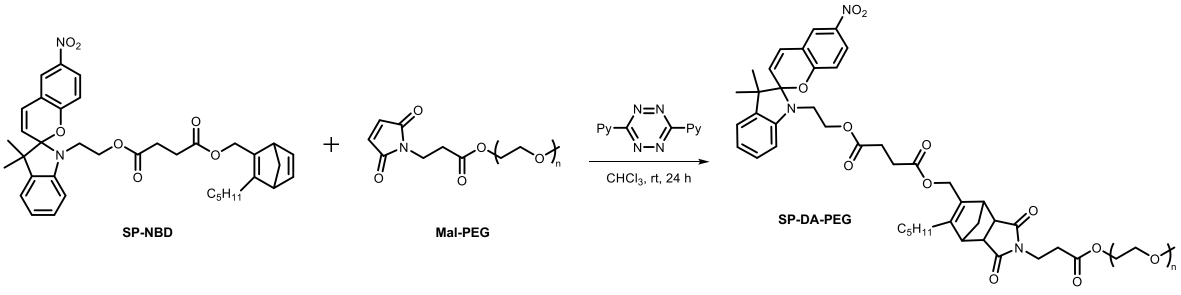

SP-DA-PEG and its precursors **SP-NBD** and **Mal-PEG** were synthesized as previously reported by Seshadri et al. (*2*). and kept under a nitrogen atmosphere at 0 °C. Spectral data matches those reported in the literature (*2*, *3*).
^{1}H NMR (500 MHz, $CDCl_3$) δ 8.02–8.00 (m, 2H), 7.20 (t, 1H), 7.08 (d, 1H), 6.93 (dd, 1H), 6.89 (t, 1H), 6.75 (d, 1H), 6.67(d, 1H), 5.89 (d, 1H), 4.68 (dd, 1H), 4.30 (dd, 1H) 4.27–4.19 (m, 4H), 3.64 (m, 47H), 3.55–3.53 (m, 2H), 3.38 (s, 3H), 2.63–2.54 (m, 4H), 2.48–2.45 (m, 2H), 2.19–2.14 (m, 1H), 1.82–1.77 (m, 1H), 1.71 (d, 1H), 1.50–1.47 (m, 2H) 1.37–1.18 (m, 9H), 1.17 (s, 3H), 0.87 (t, 3H) ppm. M_n(NMR) = 1390 g/mol.

2. Synthesis of merocyanine MCH-para:

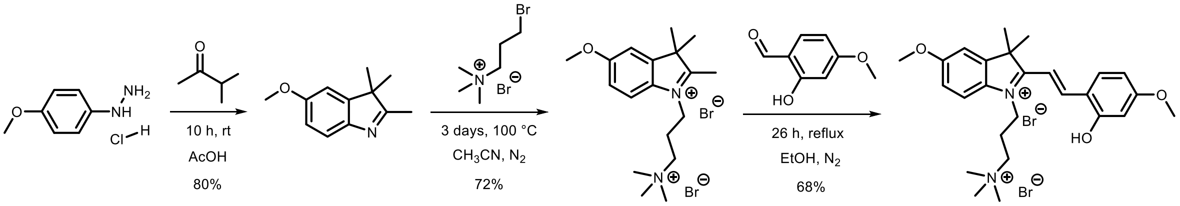

5-Methoxy-2,3,3-trimethyl-3H-indole

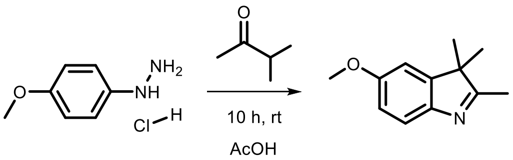

Synthesis of 5-Methoxy-2,3,3-trimethyl-3H-indole was adapted from Wimberger et al (*1*). To a solution of 3-methyl-2-butanone (2.48 mL, 2.0 g, 23.2 mmol, 2.00 eq.) in 50 mL of glacial acetic acid was added 4-methoxyphenylhydrazine hydrochloride (2.0 g, 11.6 mmol, 1.00 eq.). The mixture was heated to 140 °C for 20 min to dissolve 4-methoxyphenylhydrazine hydrochloride completely. The purple solution was stirred at room temperature overnight, after which potassium hydroxide pellets were added slowly to neutralize the mixture. The solution was extracted with diethyl ether (3 × 50 mL), and the combined organic phases were washed with brine (35 mL), dried over magnesium sulfate, and concentrated under reduced pressure. The final product can be purified by column chromatography (hex/EtOAc = 7/3 → 1/1) if necessary. 1.76 g (80% yield). Due to possible decomposition at room temperature, the product was kept in the fridge.

^{1}H NMR (500 MHz, $CDCl_3$) δ 7.43 (dd, J = 8.2, 0.7 Hz, 1H), 6.87 – 6.78 (m, 2H), 3.83 (s, 3H), 2.24 (s, 3H), 1.28 (s, 6H) ppm.
Spectral data matches the reported literature (*1*).

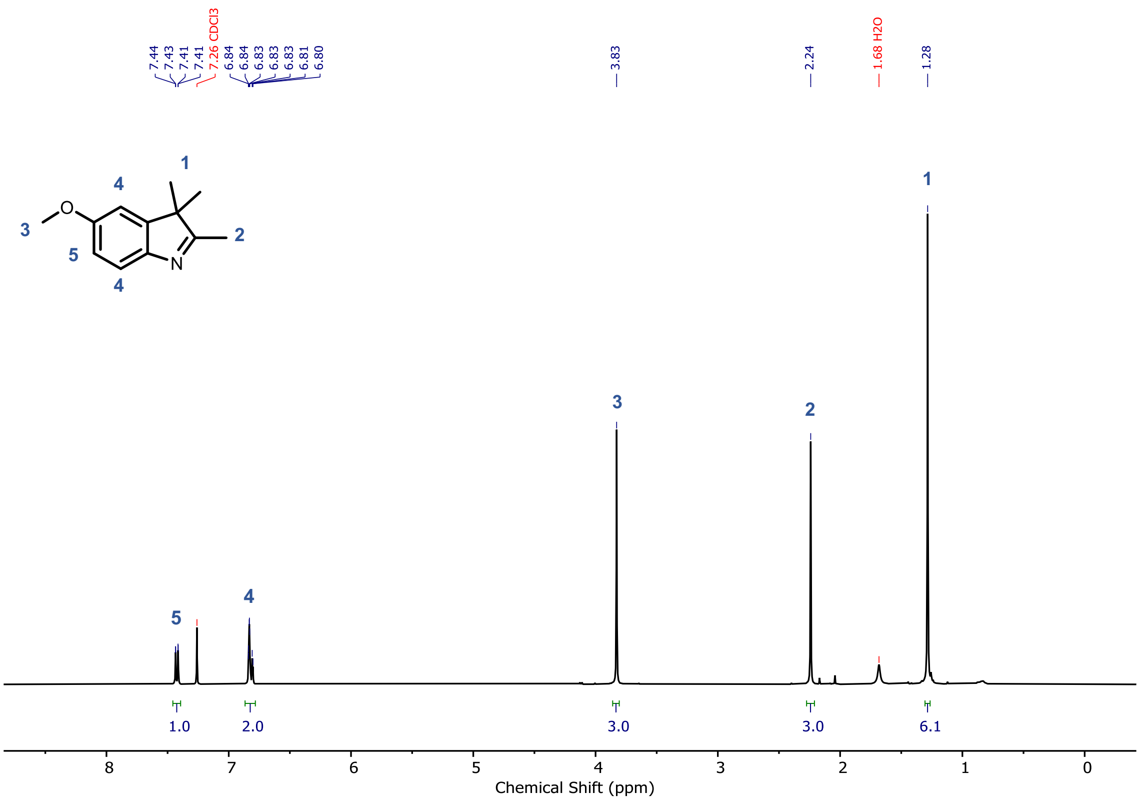

Figure S3: ^{1}H NMR spectrum of 5-Methoxy-2,3,3-trimethyl-3H-indole.

5-Methoxy-2,3,3-trimethyl-1-(3-(trimethylammonio)propyl)-3H-indol-1-ium dibromide

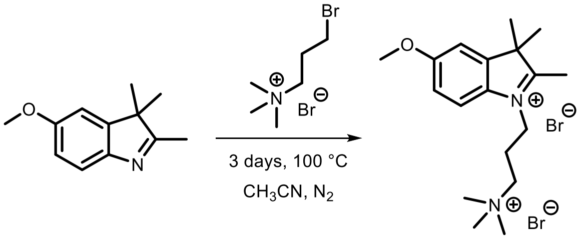

Synthesis of 5-Methoxy-2,3,3-trimethyl-1-(3-(trimethylammonio)propyl)-3H-indol-1-ium dibromide was adapted from Wimberger et al (*1*). To an oven-dry round bottom flask equipped with a magnetic stir bar, a condenser was added (3-bromopropyl)trimethyl-ammonium dibromide (1.5 g, 5.75 mmol, 1.10 eq) followed by a 5-methoxy-2,3,3-trimethyl-3H-indole (0.99 g, 5.22 mmol, 1.00 eq.) in deoxygenated absolute acetonitrile (25 mL). The reaction mixture was heated to 100 °C and refluxed under a nitrogen atmosphere for three days. After cooling down to room temperature, acetonitrile was removed under reduced pressure. The resulting purple solid was dissolved in 70 ml of water and washed with diethyl ether (3 × 40 mL) to remove impurities. The aqueous phase was evaporated in a vacuum, and the product was dissolved in a minimum amount of methanol and precipitated in cold diethyl ether, filtered, and dried in a vacuum oven overnight. The final product contains a small amount of (3-bromopropyl)trimethyl-ammonium dibromide, which due to similar solubility, was impossible to remove. Therefore, it was used without further purification. 1.69 g (72% yield).

^{1}H NMR (500 MHz, DMSO-d_6:) δ 7.99 (d, J = 8.9 Hz, 1H), 7.51 (d, J = 2.5 Hz, 1H), 7.17 (dd, J = 8.9, 2.5 Hz, 1H), 4.46 (t, J = 8.0 Hz, 2H), 3.87 (s, 3H), 3.60 – 3.57 (m, 2H), 3.11 (s, 9H), 2.85 (s, 3H), 2.32 (qt, J = 7.9, 4.9 Hz, 2H), 1.54 (s, 6H) ppm.

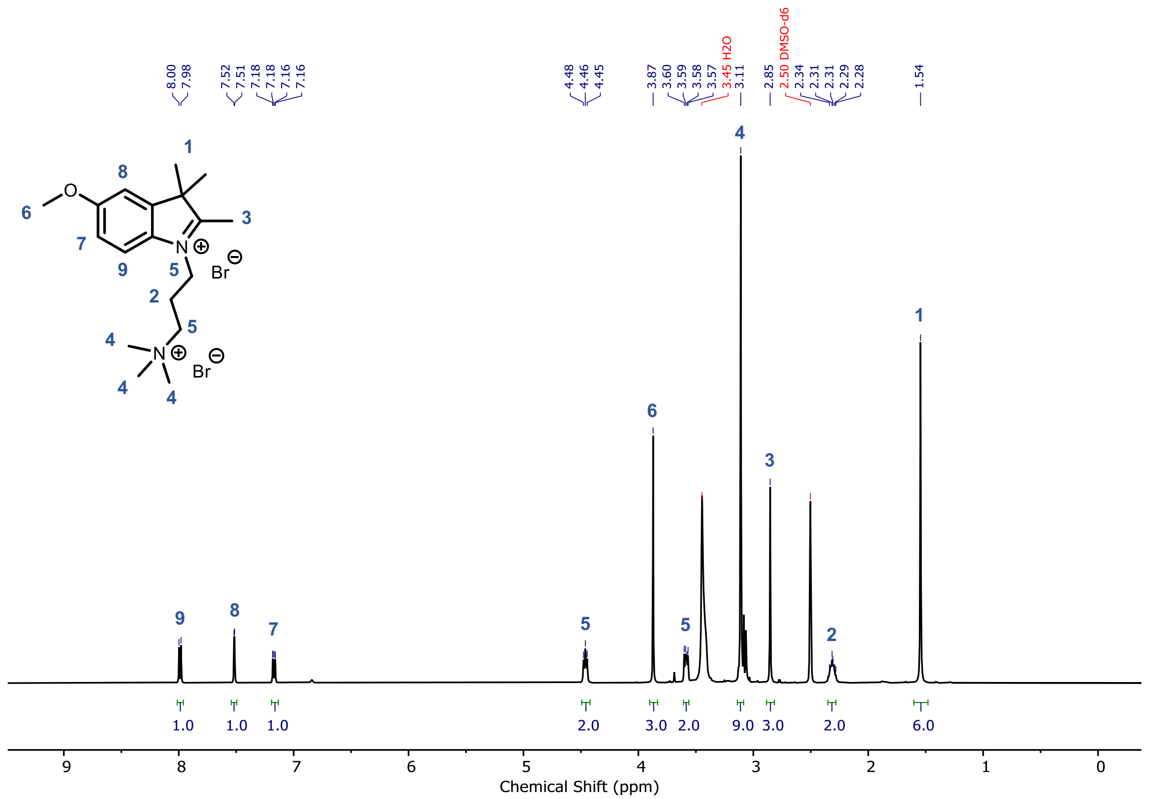

Figure S4: ^{1}H NMR spectrum of 5-Methoxy-2,3,3-trimethyl-1-(3-(trimethylammonio)propyl)-3H-indol-1-ium dibromide.

(E)-2-(2-hydroxy-4-methoxystyryl)-5-methoxy-3,3-dimethyl-1-(3-(trimethylammonio)propyl)-3H-indol-1-ium dibromide) (MCH-para)

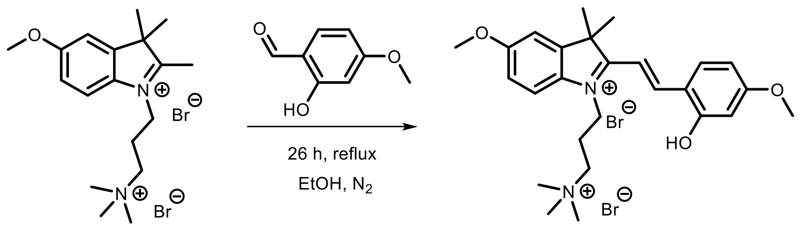

To an oven-dry three-neck round-bottom flask equipped with a magnetic stir bar and condenser was added 2-hydroxy-4-methoxybenzaldehyde (0.30 g, 1.96 mmol, 1.4 eq.) to a solution of 5-methoxy-2,3,3-trimethyl-1-(3-(trimethylammonio)propyl)-3H-indol-1-ium dibromide (0.7 g, 90 wt%, 1.40 mmol, 1.00 eq.), in 10 mL of anhydrous ethanol under nitrogen atmosphere. The reaction mixture was heated to 100 °C and refluxed under an inert atmosphere for 26 hours. After letting the solution cool down to room temperature, it was precipitated in cold diethyl ether and washed with a considerable amount of cold dichloromethane or acetone to remove the excess amount of 2-hydroxy-4-methoxybenzaldehyde. 0.52 g (68% yield).

Merocyanine form:

^{1}**H NMR** (500 MHz, DMSO-d_6:) δ 11.25 (s, 1H), 8.49 (d, J = 16.0 Hz, 1H), 8.20 (d, J = 8.9 Hz, 1H), 7.87 (d, J = 8.9 Hz, 1H), 7.53 (d, J = 2.5 Hz, 1H), 7.47 (d, J = 16.1 Hz, 1H), 7.16 (dd, J = 8.8, 2.5 Hz, 1H), 6.64 (dd, J = 8.9, 2.5 Hz, 1H), 6.60 (d, J = 2.4 Hz, 1H), 4.57 (t, J = 7.8 Hz, 2H), 3.89 (s, 3H), 3.85 (s, 3H), 3.64 – 3.58 (m, 2H), 3.11 (s, 9H), 2.28 (td, J = 10.1, 6.0 Hz, 2H), 1.76 (s, 6H) ppm.

^{13}C NMR (126 MHz, DMSO-d_6:) δ 179.63, 165.90, 161.45, 160.31, 147.71, 145.10, 134.07, 132.04, 115.65, 115.17, 114.65, 108.94, 108.23, 108.13, 100.67, 62.06, 56.20, 55.76, 52.55, 51.56, 43.11, 26.83, 21.73 ppm. TOF MS ES$^+$ exact mass calculated for $C_{26}H_{36}N_2O_3{}^{2+}$ is 212.1363, found: 212.1357

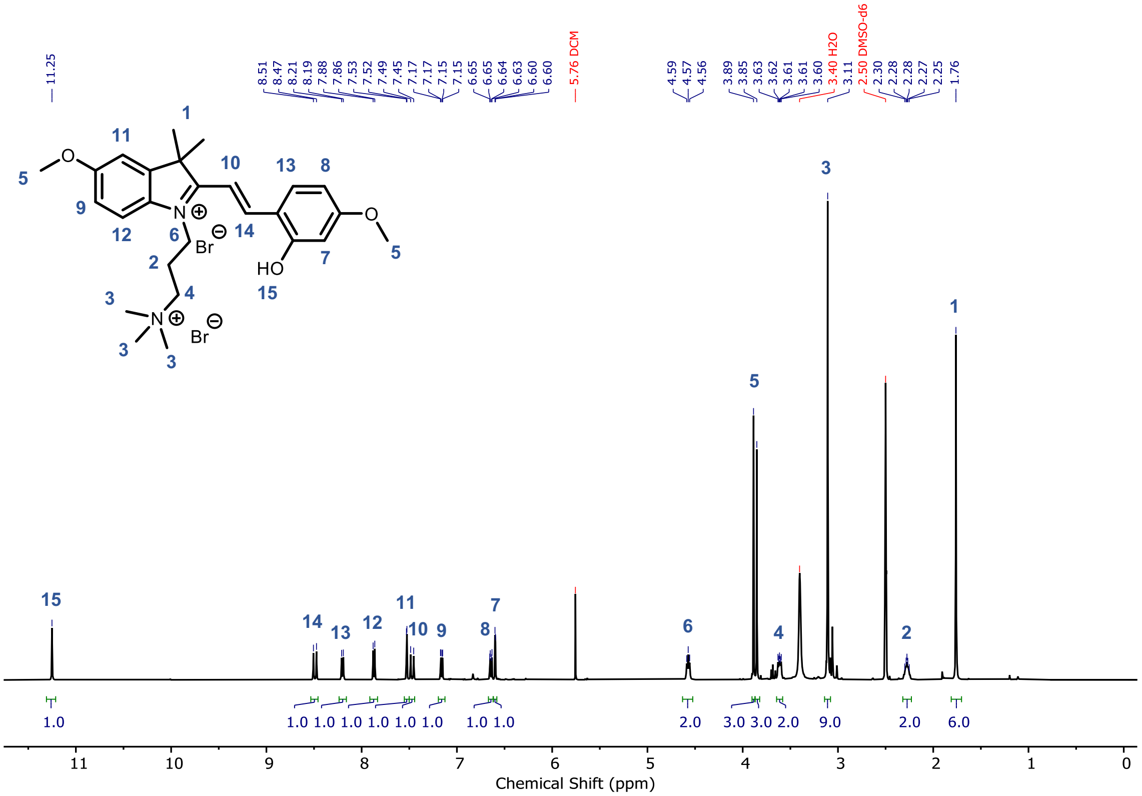

Figure S5: ^{1}H NMR spectrum of 5-Methoxy-2,3,3-trimethyl-1-(3-(trimethylammonio)propyl)-3H-indol-1-ium dibromide.

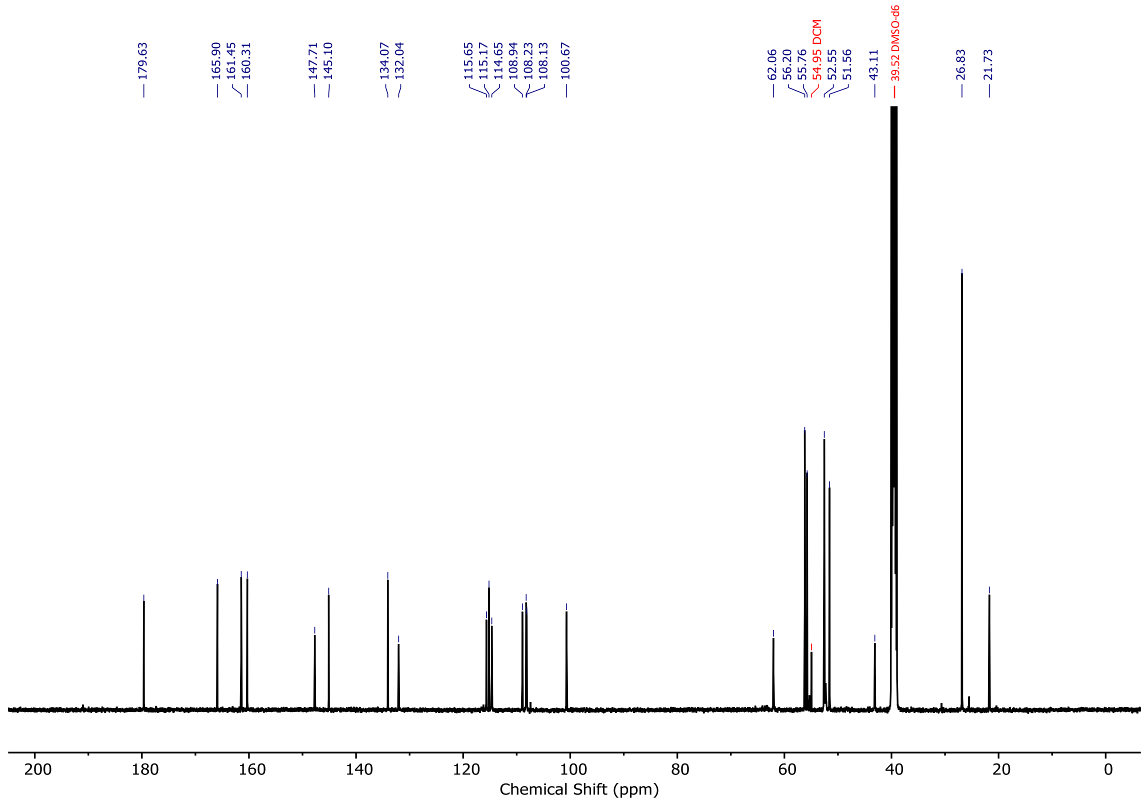

Figure S6: ^{1}H NMR spectrum of 5-Methoxy-2,3,3-trimethyl-1-(3-(trimethylammonio)propyl)-3H-indol-1-ium dibromide.

Stability of SP-DA-PEG and MCH-para in aqueous solutions

The stability of SP-DA-PEG and MCH-para is estimated by recording UV–Vis absorption spectra with an Agilent 8453 UV–Vis spectrometer and measuring surface tension with a commercial tensiometer (Theta Flex, Biolin Scientific). The 0.05 mM SP-DA-PEG solution was prepared as outlined in the methods and stored in a cool, dark environment. Weekly measurements of its UV–Vis spectra are depicted in SI Appendix, Figure S7A. After eight weeks, the SP peak shifts and decreases, indicating hydrolysis-induced degradation. Additionally, using the standard pendant drop method, the change in surface tension of a fresh SP-DA-PEG aqueous solution was measured in an inert gas, nitrogen. As shown in Figure S7B, the peak decreases as the exposure time increases, indicating that excessive exposure can hasten degradation.

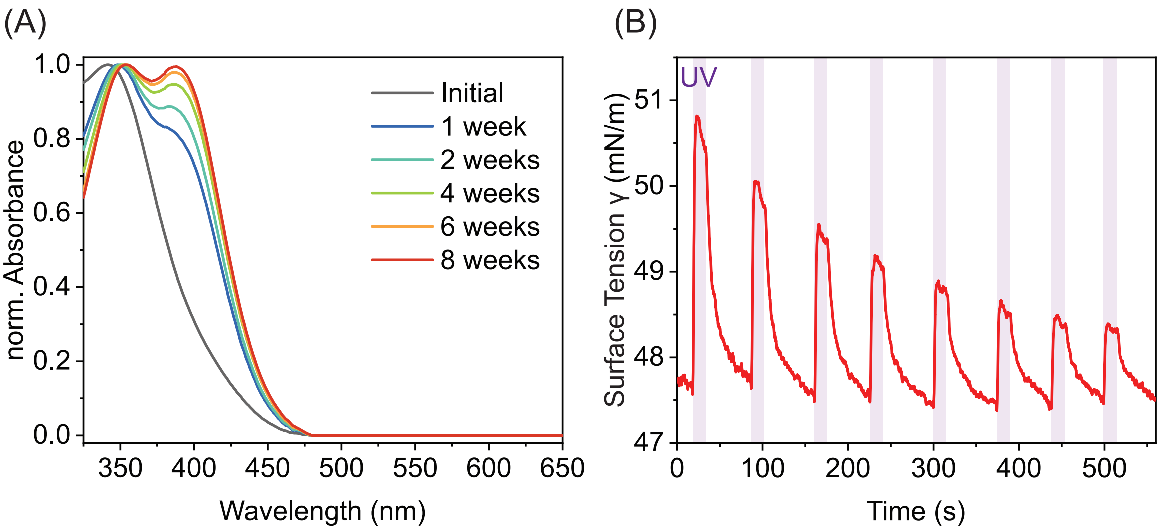

Figure S7: (A) UV-vis absorbance of SP-DA-PEG in water at 0.05 mM over eight weeks. (B) Surface tension of a solution of 0.2 mM SP-DA-PEG under repeatedly pulsed UV light.

MCH-para is stable in acidic aqueous media at room temperature for more than one month. Spectra of UV–Vis absorbance for 1 mM solutions are recorded. In Figure S8A, UV-Vis absorbance spectra with a 1–10 pH range indicate that both MC and MCH states can exist in a solution depending on its acidity. The equilibrium shifts to the MCH state at pH values between 1 and 4 (Figure S8A). MCH is stable at pH ranges between 1 and 3, as shown in Figure S8B, where peak decreases are all less than 10% after six weeks.

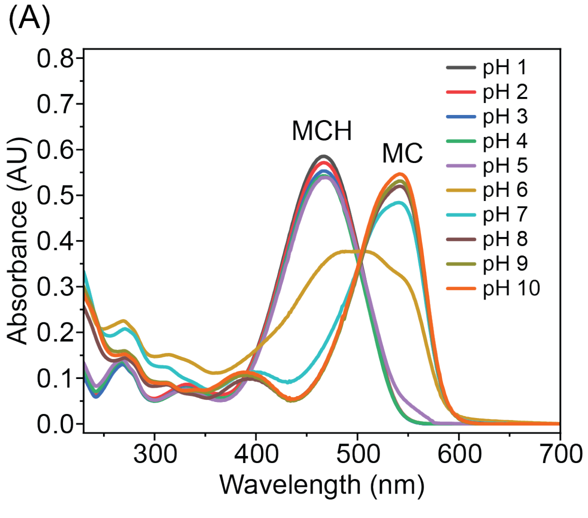

Figure S8: (A) UV-Vis absorbance spectra of equilibrated coexisting states of MCH-para aqueous solution over the pH range of 1 to 10. MCH form is the predominant form in low pH solutions, whereas MC is the predominant form in high pH solutions. (B) UV-Vis spectra of trans-MCH para in low pHs. Red line and blue line overlap.

Switching effect of SP-DA-PEG and MCH-para

Surfactants are amphiphilic molecules that are typically classified by their hydrophobic and hydrophilic parts. Our hypothesis is that surface tension correlates with hydrophilicity, which is influenced by the polarity of the molecules. For SP-DA-PEG, the transition to the merocyanine form involves a shift from the less polar spiropyran to the more polar *trans*-merocyanine, which makes the molecules have a stronger affinity for water due to electrostatic interactions with water molecules. Consequently, this increase in hydrophilicity causes the molecules to be less likely to accumulate at the interface but more dispersed in the bulk water, helping to maintain the cohesion between water molecules at the interface and sustain a high surface tension value. Similarly, the surface tension decreases when MCH-para switches from the more hydrophilic *trans*-form to the less hydrophilic *cis*-form. Regarding the orientation of the molecules accumulated at the interface, we hypothesize that charged polar alkylammonium and hydroxy groups will be oriented toward the aqueous phase, while non-polar aromatic groups will be aligned toward the Krytox phase. Further investigation may be acquired by Cryo-EM in future studies.

In general, spiropyran is a photoswitchable molecule that changes under UV-light irradiation to Merocyanine form (MCH/MC, depending on conditions the molecule will be either protonated/deprotonated). When the molecule has an electron-withdrawing -NO_2 group, the equilibrium is shifted to the non-polar Spiropyran (SP) form in the dark (*4*, *5*); however, with the introduction of electron-donating methoxy groups to the light-responsive molecule, the molecular equilibrium is shifted to the more polar MCH/MC) form (*1*). So, in the case of photosurfactant on the left (Figure 1D), we switch between less polar Spiropyran and more polar *trans*-Merocyanine (*trans*-MC). In the second case, the molecule switches between more polar *trans*-Merocyanine (*trans*-MCH-para) and less polar *cis*-Merocyanine (*cis*-MCH-para). Previously, the *cis*-Merocyanine molecule (*6*) was attributed to protonated Spiropyran (SPH). However, it has been recently demonstrated (*7*) that it is more correct to refer to it as *cis*-Merocyanine (*cis*-MCH), which behaves similarly to SPH due to the apparent barrierless transition from SP to *cis*-MCH at low pH.

Temperature rise of droplet due to heating from light illumination

It is possible for the thermal Marangoni effect induced by irradiating the substances with light to partially contributes to the droplet motion. Surface tension of an aqueous solution containing the photo-responsive surfactants as a function of temperature was measured using the standard pendant drop method on a commercial tensiometer. The increase in temperature caused by illumination of light with specific intensity was estimated using an infrared (IR) camera (Telops M3K). To decouple the photo-Marangoni effect from heating effect under illumination, we first heated the solution to varying temperatures using a custom syringe heating system and then measured the surface tension. Several film heater plates were attached to the exterior of the syringe to heat the solution, and a thermocouple was attached to the needle to measure the temperature of the injected droplet (Figure S9A). As a result, the relationship between surface tension and temperature is linear, ranging from 20°C to 60°C (Figure S9B). As temperature increases, surface tension decreases:

$$\gamma \left[\frac{\mathrm{mN}}{\mathrm{m}}\right] = -0.26\, T\, [^\circ\mathrm{C}] + 76.66. \tag{1}$$

To further assess the thermal effect, a series of temperature measurement experiments have been conducted. The emissivity of an aqueous solution containing 1 mM MCH-para was calibrated with reference to the emissivity of deionized water. Images of the two droplets show comparable temperature profiles and heat distributions when two identically sized droplets of deionized water and MCH-para solution are subjected to the same room temperature and light conditions (Figure S9C). As a result, the emissivity of water, $\epsilon = 1$, can be used to estimate the emissivity of MCH-para. The temperature rise in MCH-para droplet due to blue LED illumination with an intensity of 31.8 mW/cm^2 is small. The collimated 470 nm LED used to illuminate the droplet is the same one utilized for measuring surface tension (Figure S10A). As comparison, the LED was first used to heat a water droplet of equal size, resulting in a temperature change of less than 1 °C (Figure S10B). Then, the MCH-para droplet was stabilized at room temperature. After 150 s of heating, the maximum temperature within the droplet rises from 19.6 °C to 20.6 °C (Figure S10C-D), resulting in a 0.26 mN/m decrease in surface tension. This decrease is negligible compared to the total change of 4.4 mN/m in surface tension (pH = 3) depicted in Figure 1H. The thermal effect caused by irradiation only accounts for 5.9% of the total change in surface tension. For blue light with similar intensities, the photo Marangoni effect is the primary factor that induces shear flow and causes droplet motions.

In the case of SP-DA-PEG, UV illumination has been observed to cause an increase in both surface tension and interfacial tension. Even this suggests that the direction of the photo-Marangoni stress must be reversed to overcome thermal influences and lead to movement away from the light source, it is essential to study how much the temperature increase when exposed to UV light. This study helps to figure out how to make bigger changes in surface tension by reducing thermal Marangoni effect that could interfere with this process. We utilized a T-type thermocouple probe to measure the temperature increase in the aqueous droplet. The experimental set-ups for both UV LED and laser illumination experiments are depicted in Figure S11. In these experiments, we placed a equal-sized droplet onto the surface. Uniform illumination was ensured on the droplet, preventing any movement across the surface during the procedure. For data precision, temperature readings were taken at 0.5-second intervals using a data acquisition system (Keysight DAQ973A).

In Figure S12A, we observe that the temperature rises and stabilizes after an increase of approximately 1.8 °C over 100 seconds. This stabilization is attributable to the relatively lower power of the UV LED in comparison to the UV laser. Thus, it is important to note that the migration of droplets under the UV laser is still influenced by thermal effects. This was further investigated using the same type of thermocouple probe to record the temperature changes, as depicted in Figure S12B. Upon 50 seconds of direct exposure to UV heating, there was a measured increase in temperature by 3.1 °C. This modest rise was insufficient to induce a change greater than 1 mN/m in surface tension, referencing the response of water under varying temperatures (*8*). The minimal fluctuation can be ascribed to the effective thermal conductivity of the silicon surface, which was positioned on an optical table with a metallic surface, facilitating heat dissipation.

Figure S12C-D in the study present data concerning the temperature rise in a droplet floating on liquid lubricant under different conditions. In Figure S12C, the droplet experiences a temperature increase of 5.9 °C. However, this increase is also minimal and insufficient to bring about a reduction in surface tension of 1 mN/m. This observation underscores the relatively subdued impact of temperature

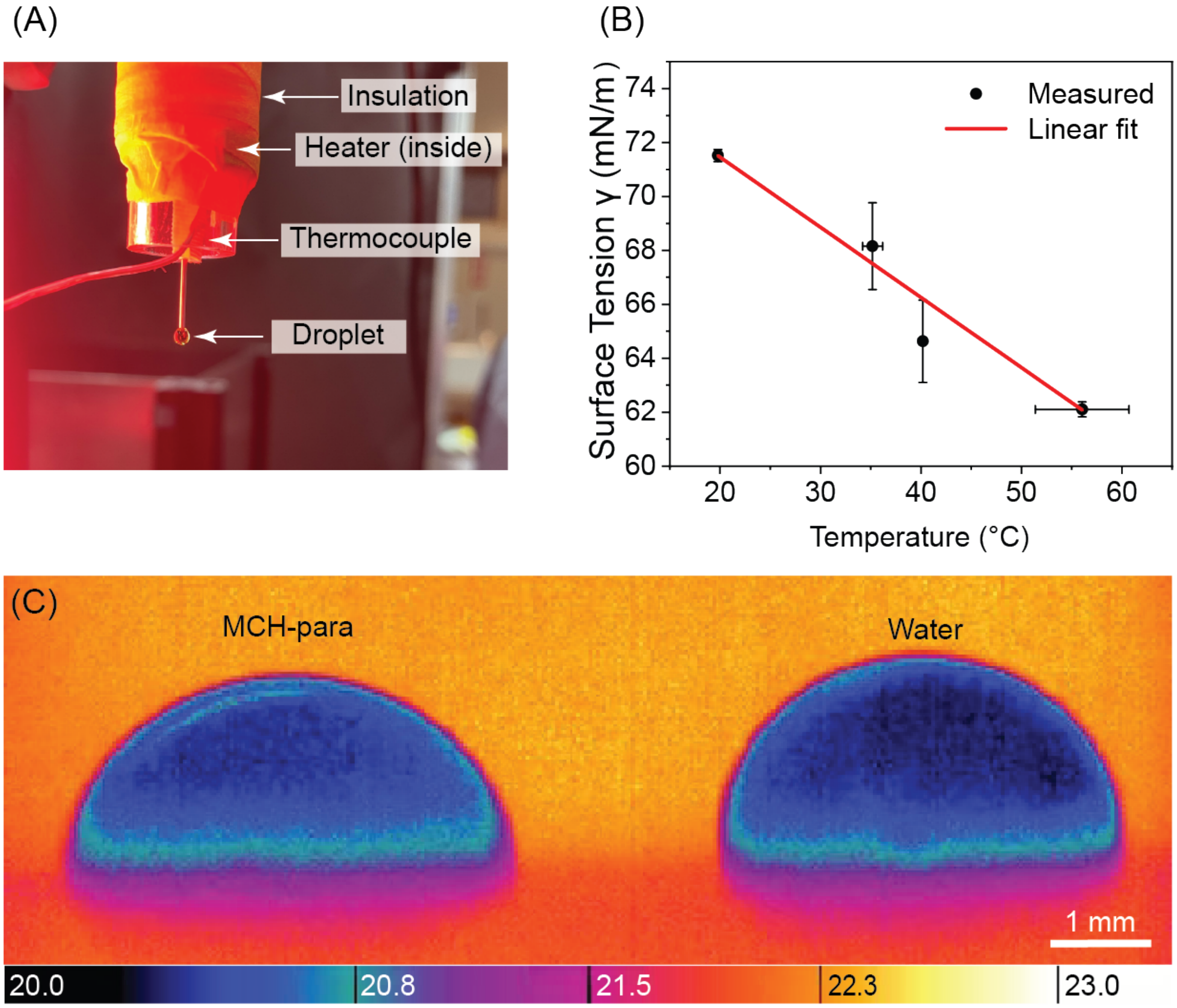

Figure S9: (A) Experimental setup for measuring surface tension at various temperatures. (B) The response of MCH-para surface tension to temperature. (C) IR images of the deionized water (right) and the MCH-para aqueous solution droplet (left; pH = 3) at room temperatrue. Ranges on the legend bar represent temperatures in °C.

on altering surface tension in this configuration. Figure S12D simulated scenario corresponding to the conditions in Figure 4B, where the droplet is subjected to a pulsed laser. The laser's operation alternates between 'on' and 'off' states every 30 seconds to investigate the cumulative temperature effect. After five heating cycles, the accumulated heat contributes to a temperature elevation of approximately 4.0 °C.

From these observations, it becomes evident that despite the presence of the heating from light, its influence on the aqueous droplets with SP-DA-PEG is trivial. The more significant factor appears to be the photo-Marangoni effect, attributable to the compositional gradient within the droplet.

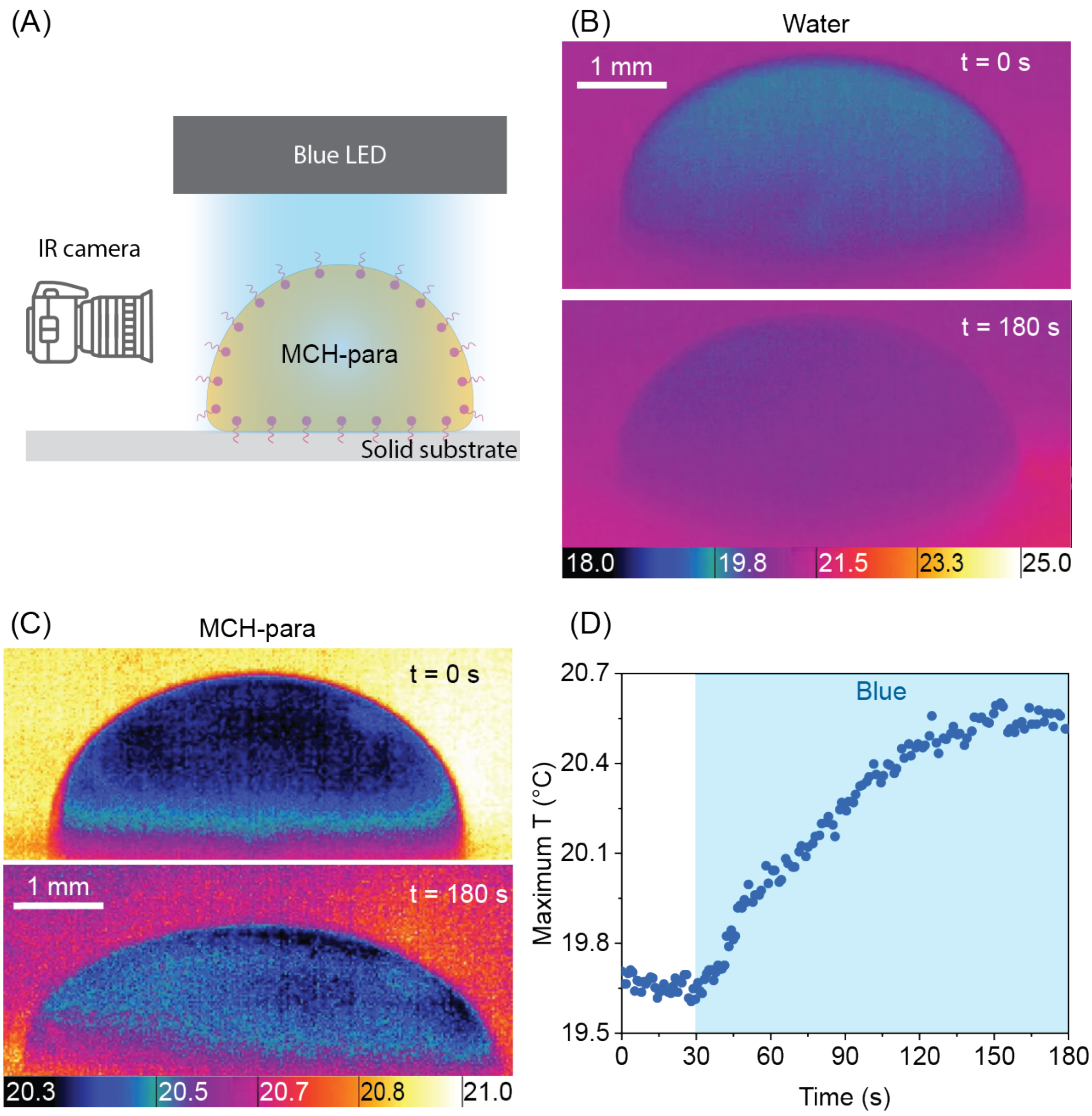

Figure S10: (A) Schematic of single droplet IR image capture under collimated LED illumination (31.8 mW/cm^2). (B) IR images of blue light-heated deionized water droplets. (C) IR images of 1 mM MCH-para water droplet heated by blue light illuminations. (D) Maximum temperature occurs within the MCH-para droplet over six minutes.

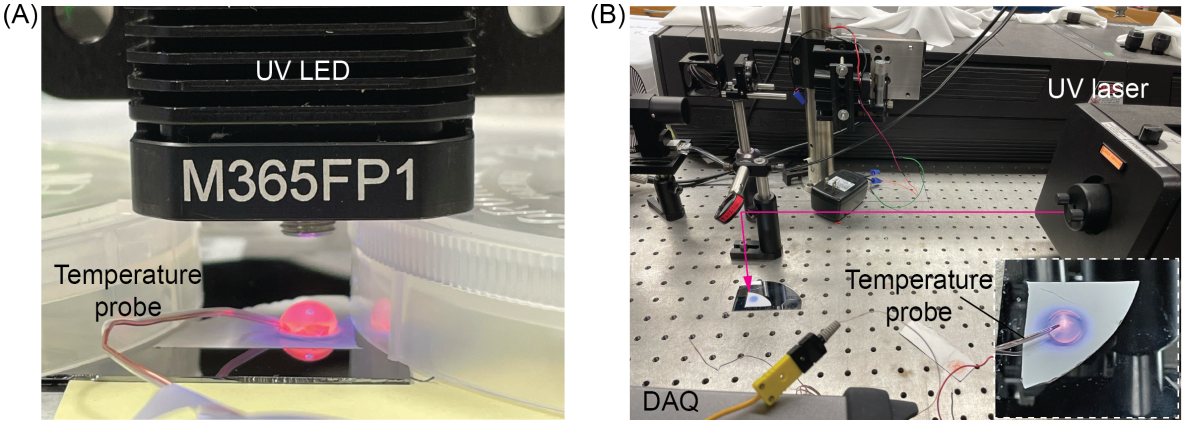

Figure S11: Measuring the temperature rise of 0.2 mM aqueous solution due to UV LED and laser heating. (A) Experimental set-up of UV LED heated aqueous droplet. (B) Experimental set-up of UV laser-heated aqueous droplet.

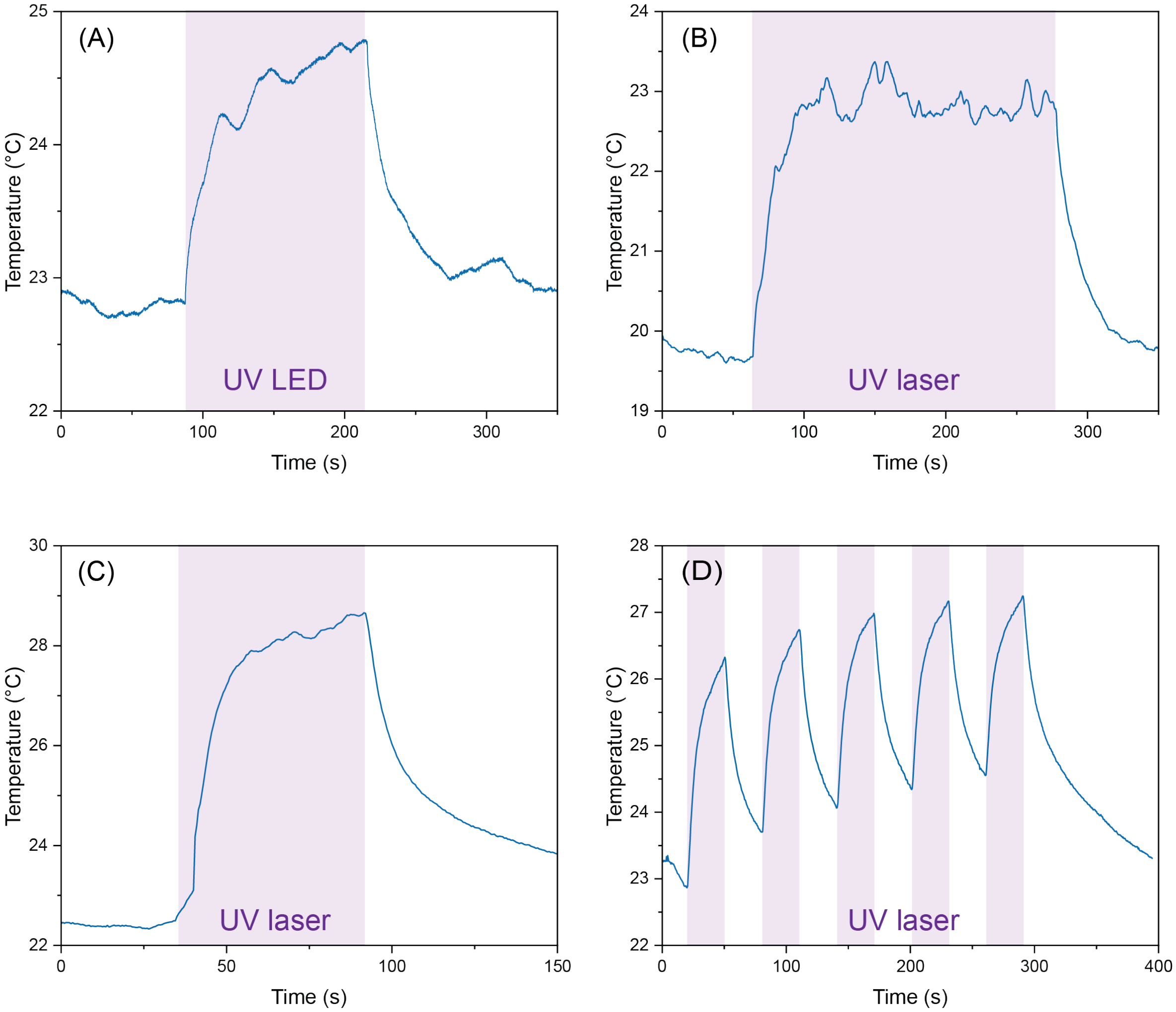

Figure S12: Temperature rise of 0.2 mM aqueous droplet on silicon wafer (A) upon UV LED illumination. (B) upon UV laser illumination. Temperature rise of 0.2 mM aqueous droplet on lubricant (C) upon UV laser illumination. (D) upon pulsed UV laser.

Simulations of drops over liquid-infused surfaces

In order to help understand the flow patterns arising within drops on LIS, we performed simulations using COMSOL Multiphysics. The simulations solved the Navier-Stokes equations for the transport of mass and momentum in the fluid. For simplicity, the aqueous drop shape is approximated as a hemisphere, and is placed on an oil layer of uniform thickness. The flow is assumed steady in a reference frame moving at a velocity V with drop. Therefore, at the oil-water interface we enforce no normal flow, and we enforce uniform flow, equal to $-V$, in the oil layer sufficiently far away from the drop. The bottom of the oil layer has a no-slip boundary condition in the fixed reference frame, and therefore has a velocity equal to $-V$ when observed in the frame of reference moving with the drop. We enforce continuity of tangential velocity at the drop-oil interface. The effect of the Marangoni stress is captured by applying a meridional stress along the bottom and top boundaries of the drop. A refined mesh at the interface and near the endpoints is used to ensure numerical accuracy.

Scaling theory for drops in liquid

The Marangoni stress across the drop is of order $(\gamma_{\rm hi} - \gamma_{\rm lo})/R$, where $\gamma_{\rm hi}$ and $\gamma_{\rm lo}$ are the interfacial tension values at the front and rear of the drop, whereas R is the drop radius. The drop surface area is of order R^2; therefore

$$F_{\rm Ma} \sim (\gamma_{\rm hi} - \gamma_{\rm lo})R. \tag{2}$$

At low Reynolds numbers $Re = \rho V 2R/\mu$ (where ρ, μ are the Krytox density and viscosity), the fluid resistance associated with a self-propelled drop, moving through a fluid whose viscosity is much greater than the drop's, is of order $F_{\rm drag} \sim \mu V R$ (*9*). Therefore the balance $F_{\rm drag} \sim F_{\rm Ma}$ yields

$$V \sim \frac{\gamma_{\rm hi} - \gamma_{\rm lo}}{\mu} \quad \text{if} \quad \mu \gg \mu_{\rm drop}, Re \ll 1. \tag{3}$$

We must determine how the difference $\gamma_{\rm hi} - \gamma_{\rm lo}$ depends on other parameters, such as drop radius. Let us label $\gamma_{\rm A}$ the tension in the rest (dark) state, whereas $\gamma_{\rm B}$ is the steady-state value eventually reached under illumination. Empirically, based on our surface tension measurements, we approximate the forward switch dynamics (starting in the dark and illuminating at $t = 0$)

$$\gamma(t) = \gamma_{\rm B} + (\gamma_{\rm A} - \gamma_{\rm B})\, {\rm e}^{-t/\tau_{\rm fwd}}, \tag{4}$$

where $\tau_{\rm fwd}$ is the timescale for the forward switch. Similarly, when illumination is stopped from the initial steady state $\gamma = \gamma_{\rm B}$, the interfacial tension follows approximately

$$\gamma(t) = \gamma_{\rm A} + (\gamma_{\rm B} - \gamma_{\rm A})\, {\rm e}^{-t/\tau_{\rm rev}}, \tag{5}$$

where $\tau_{\rm rev}$ is the timescale for the reverse switch. Here, $\gamma_{\rm A}, \gamma_{\rm B}, \tau_{\rm rev}$ and $\tau_{\rm fwd}$ are found empirically from surface-tension measurements, such as those in Figure S7B.

To find expressions for $\gamma_{\rm hi}$ and $\gamma_{\rm lo}$, we derive a set of equations relating the interfacial tensions at key locations on the drop. We label the solution the leaves the illuminated region of the drop as $\gamma_{\rm hi}$. This liquid takes a time $t_{\rm d} \sim (2R - d)/V_{\rm I}$ to reach the rear end of the drop, where $V_{\rm I}$ is the fluid velocity scale at the interface and in the interior of the drop. Therefore, noting that the initial condition is $\gamma = \gamma_{\rm hi}$, Eq. (5) becomes:

$$\gamma_{\rm lo} \sim \gamma_{\rm A} + (\gamma_{\rm hi} - \gamma_{\rm A})\, {\rm e}^{-t_{\rm d}/\tau_{\rm rev}} \tag{6}$$

where d is the light beam diameter. As the liquid recirculates inside the drop, the photo-surfactant continues its reverse reaction. Even though this liquid is not at an interface, we can model the value of the interfacial tension that would arise if this fluid element were placed at an interface (for brevity, we label this a 'virtual tension'). Because the liquid has traveled over a time of order $2t_{\rm d}$ since it was last illuminated, as it enters the illuminated region again it will have a virtual tension, labeled γ_1,

$$\gamma_1 \sim \gamma_{\rm A} + (\gamma_{\rm hi} - \gamma_{\rm A})\, {\rm e}^{-2t_{\rm d}/\tau_{\rm rev}}. \tag{7}$$

The highest surface tension is reached by illumination of this fluid as it travels a length of order $2d$, over a time $t_{\rm i} \sim 2d/(V_I \tau_{\rm rev})$,

$$\gamma_{\rm hi} \sim \gamma_{\rm B} + (\gamma_1 - \gamma_{\rm B})\, {\rm e}^{-t_{\rm i}/\tau_{\rm rev}}. \tag{8}$$

We use Eqs. (6), (7) and (8) in Eq. (3) and eliminate $\gamma_1, \gamma_{\mathrm{hi}}$, and γ_{lo}, obtaining:

$$V_{Re\ll 1} \sim \frac{\gamma_{\mathrm{hi}} - \gamma_{\mathrm{lo}}}{\mu} \sim \frac{\gamma_{\mathrm{B}} - \gamma_{\mathrm{A}}}{\mu} \frac{(1 - \mathrm{e}^{-t_{\mathrm{d}}/\tau_{\mathrm{rev}}})(1 - \mathrm{e}^{-t_{\mathrm{i}}/\tau_{\mathrm{fwd}}})}{1 - \mathrm{e}^{-(2t_{\mathrm{d}}/\tau_{\mathrm{rev}} + t_{\mathrm{i}}/\tau_{\mathrm{fwd}})}}. \tag{9}$$

If $Re \ll 1$, we can assume $V_I \sim V$. Introducing scaling constants c_1 and c_2, we obtain an implicit equation for V:

$$V_{Re\ll 1} = c_1 \frac{\gamma_{\mathrm{B}} - \gamma_{\mathrm{A}}}{\mu} \frac{(1 - \mathrm{e}^{-c_2 t_{\mathrm{d}}/\tau_{\mathrm{rev}}})(1 - \mathrm{e}^{-c_2 t_{\mathrm{i}}/\tau_{\mathrm{fwd}}})}{1 - \mathrm{e}^{-c_2(2t_{\mathrm{d}}/\tau_{\mathrm{rev}} + t_{\mathrm{i}}/\tau_{\mathrm{fwd}})}}. \tag{10}$$

Eq. (10) above is solved by iteration and is used to plot the dotted line in Fig. 4E. We use $\gamma_{\mathrm{B}} - \gamma_{\mathrm{A}} = 0.73\,\mathrm{mN/m}$, as this is the value observed after repeated illumination cycles (as exemplified in Fig. S7B), and $\tau_{\mathrm{fwd}} = 0.437\,\mathrm{s}, \tau_{\mathrm{rev}} = 2.19\,\mathrm{s}, \mu = 1.24 \cdot 10^{-2}/\,\mathrm{kg/(m\,s)}$, $d = 1.3\,\mathrm{mm}$ and $R = (\frac{4}{3\pi}\mathcal{V})^{1/3}$, where $\mathcal{V}$ is the drop volume. The constants we use are $c_1 = 0.2$ and $c_2 = 0.15$; we note these are of order-one, as should be expected for a physically plausible scaling theory.

It is instructive to seek an approximation of Eq. (10) for small drops. We assume that, as R approaches zero, V decreases more slowly than R, such that the ratio $R/(V_I\,\tau_{\mathrm{rev}})$ also approaches zero. Since in practice the beam diameter d is essentially constant, we assume that the ratio $d/(V_I\,\tau_{\mathrm{fwd}}) \sim t_{\mathrm{i}}/\tau_{\mathrm{rev}} \gg 1$, such that, in Eq. (10), we neglect the second exponential in the numerator, as well as the exponential in the denominator:

$$V_{Re\ll 1} \approx c_1 \frac{\gamma_{\mathrm{B}} - \gamma_{\mathrm{A}}}{\mu} (1 - \mathrm{e}^{-c_2 t_{\mathrm{d}}/\tau_{\mathrm{rev}}}). \tag{11}$$

Introducing a Taylor expansion in $t_{\mathrm{d}}/\tau_{\mathrm{rev}}$, neglecting second-order terms, and solving for V, we obtain the explicit expression for the low-Re velocity:

$$V_{Re\ll 1} \approx \sqrt{c_1 c_2 \frac{\gamma_{\mathrm{B}} - \gamma_{\mathrm{A}}}{\mu} \frac{2R - d}{\tau_{\mathrm{rev}}}}, \tag{12}$$

suggesting that for small drops the velocity is highly sensitive to volume, as $V \sim R^{1/2} \sim \mathcal{V}^{1/6}$.

At large drop volumes, we observe empirically that the velocity decreases slightly. There are several potential explanations for this observation. We tentatively consider here the effect of finite Reynolds number, as the larger drops have $Re = \rho V 2R/\mu \sim 4$. For a self-propelled object, the propulsive thrust T scales as $T \sim \dot{m}\Delta V$, where $\dot{m}$ is the mass flow rate that is accelerated by a velocity increment ΔV. Here $\dot{m} \sim \rho(V + \Delta V)\delta^2$, where δ is the half-width of the faster-moving fluid region behind the drop. Equating the thrust with the Marangoni force $\sim (\gamma_{\mathrm{hi}} - \gamma_{\mathrm{lo}})R$ we find

$$(\gamma_{\mathrm{hi}} - \gamma_{\mathrm{lo}})R \sim \rho(V + \Delta V)\Delta V \delta^2. \tag{13}$$

For large $Re \gg 1$, we use the canonical laminar flow scaling $\delta^2 \sim R^2\,Re_{\Delta V}^{-1} \sim R\nu\Delta V^{-1}$. Assuming $\Delta V \gg V$, we approximate $(V + \Delta V) \sim \Delta V$ in (13), such that the expression for $\gamma_{\mathrm{hi}} - \gamma_{\mathrm{lo}}$ simplifies to

$$(\gamma_{\mathrm{hi}} - \gamma_{\mathrm{lo}}) \sim \mu\Delta V. \tag{14}$$

To relate V and ΔV, we assume that at $Re \gg 1$ the fluid resistance follows an inertial scaling, and is therefore proportional to $\rho V^2 R^2$. Balancing this fluid resistance with the Marangoni thrust T,

$$\rho(V + \Delta V)\Delta V \delta^2 \sim \rho V^2 R. \tag{15}$$

Using again the assumption $\Delta V \gg V$, Eq. (15) reduces to

$$\Delta V \sim V^2 \frac{R}{\nu}. \tag{16}$$

Note that Eq. (16) can be rewritten to give

$$\frac{\Delta V}{V} \sim \frac{RV}{\nu} = Re \gg 1, \tag{17}$$

which supports our assumption $\Delta V \gg V$.

Finally, substituting (16) into (14), solving for V, and introducing scaling constants C_1, C_2, C_3

$$V_{Re\gg1} = C_1 \sqrt{\frac{\gamma_{\mathrm{hi}} - \gamma_{\mathrm{lo}}}{R\rho}}, \tag{18}$$

$$\gamma_{\mathrm{hi}} - \gamma_{\mathrm{lo}} = (\gamma_{\mathrm{B}} - \gamma_{\mathrm{A}}) \frac{\left[1 - \exp\left(-C_2 \frac{2R-d}{V_{\mathrm{I}}\,\tau_{\mathrm{rev}}}\right)\right]\left[1 - \exp\left(-C_2 \frac{2d}{V_{\mathrm{I}}\,\tau_{\mathrm{fwd}}}\right)\right]}{1 - \exp\left[-C_2\left(2\frac{2R-d}{V_{\mathrm{I}}\,\tau_{\mathrm{rev}}} + \frac{2d}{V_{\mathrm{I}}\,\tau_{\mathrm{fwd}}}\right)\right]}, \tag{19}$$

$$V_I = V_{Re\gg1} + C_3\, V_{Re\gg1}^2 \frac{R}{\nu}. \tag{20}$$

The expression (18) is used to plot the dashed line in Fig. 4E, with $C_1 = 1.2, C_2 = 3.9, C_3 = 0.1$. For large drops, $V_{Re\gg1} \sim R^{-1/2} \sim \mathcal{V}^{-1/6}$. A composite relation of the two Reynolds number regimes is shown by the continuous line in Fig. 4E, and is obtained using

$$V_{\mathrm{composite}} = \left[(V_{Re\ll1})^{-m} + (V_{Re\gg1})^{-m}\right]^{-1/m} \tag{21}$$

where we set $m = 12$.

Safety Statement

No unexpected or unusually high safety hazards were encountered.

Supplementary Figures

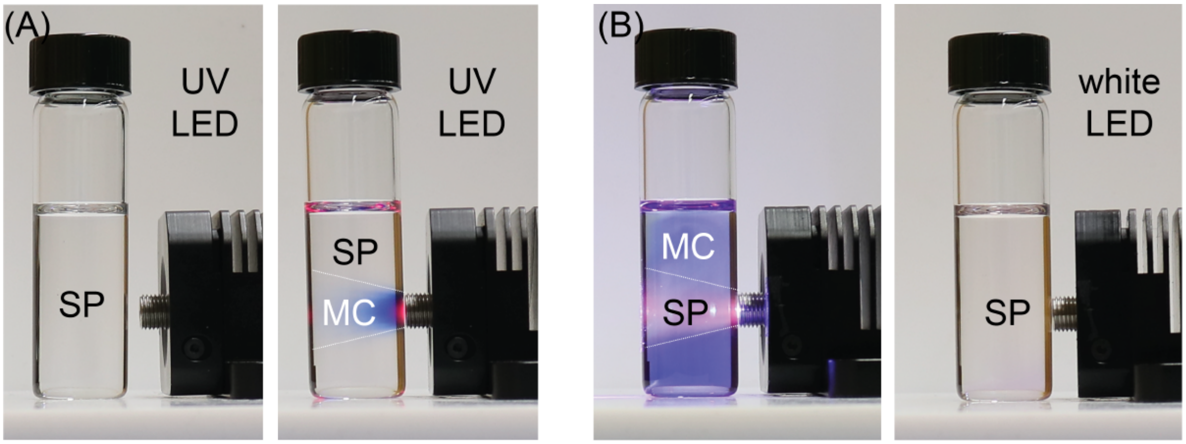

Figure S13: The changes in the color of SP-DA-PEG in toluene under UV illumination. (A) 0.1 mM SP toluene solution transforms from transparent (SP) to dark blue (MC) under 365 nm UV illumination. (B) After achieving equilibrium, the MC-rich solution under white light illumination changes color from dark blue to transparent.

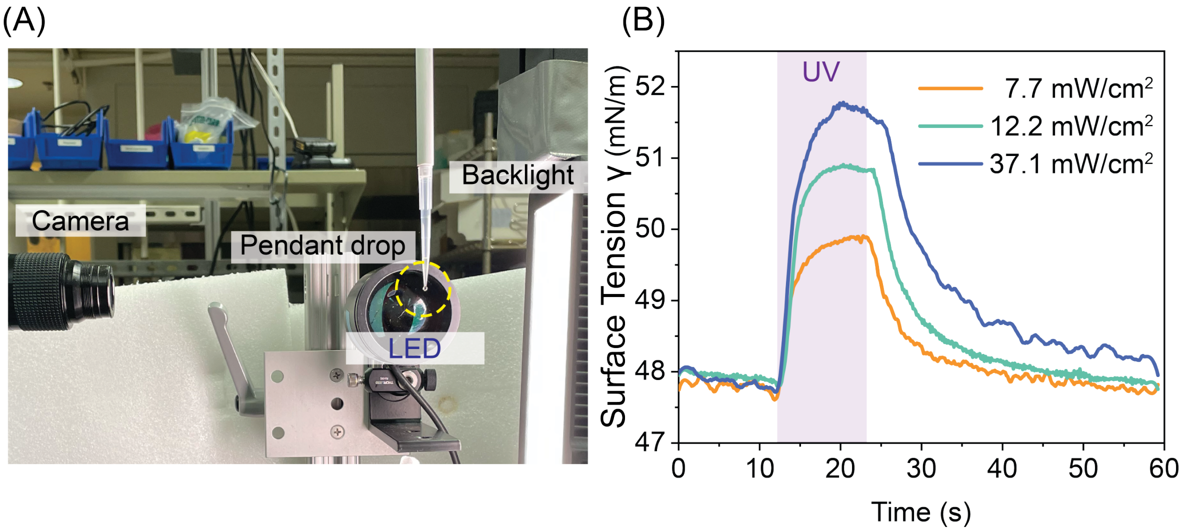

Figure S14: (A) Experimental setup for measuring surface tension under repeated exposure to corresponding light. (B) Surface tension response of 0.2 mM SP-DA-PEG in water under UV (365 nm) illumination with optical intensities in the range of 7.7 to 37.1 mW/cm^2.

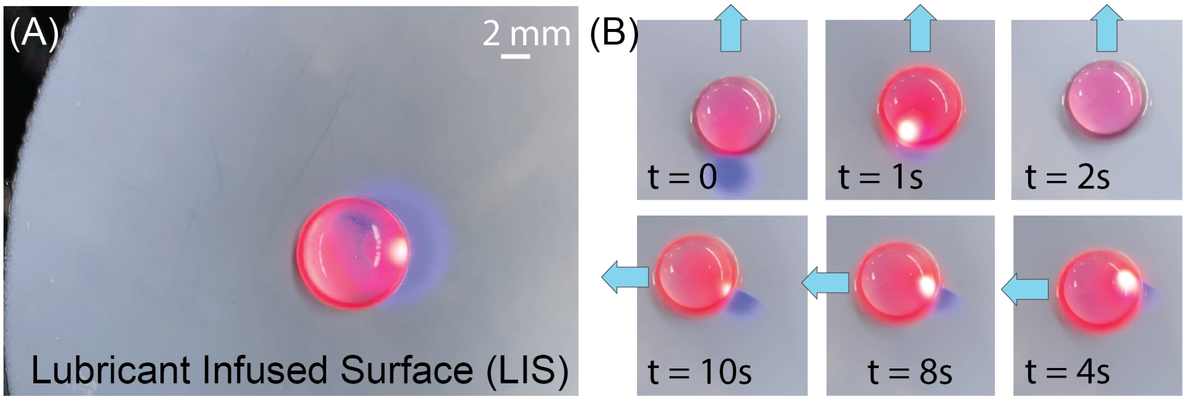

Figure S15: (A) Schematic of a liquid droplet on LIS under UV illumination. (B) Time-lapse optical images (top-down view) of water droplets containing SP-DAPEG on LIS, changing moving direction.)

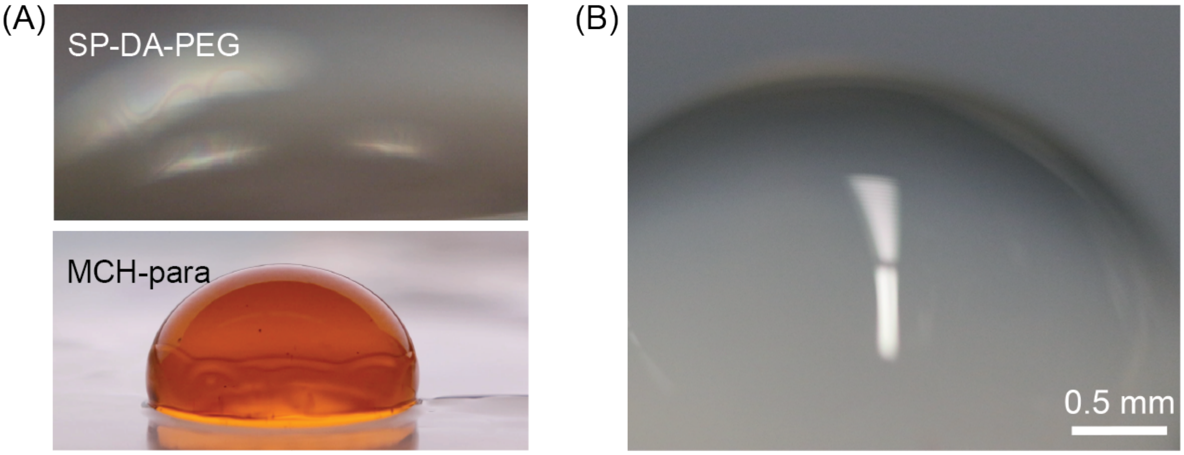

Figure S16: Surface of (A) a bare 0.2 mM SP-DA-PEG and a 1 mM MCH-para aqueous droplet placed on LIS. (B) a DI water droplet placed on the PTFE film.

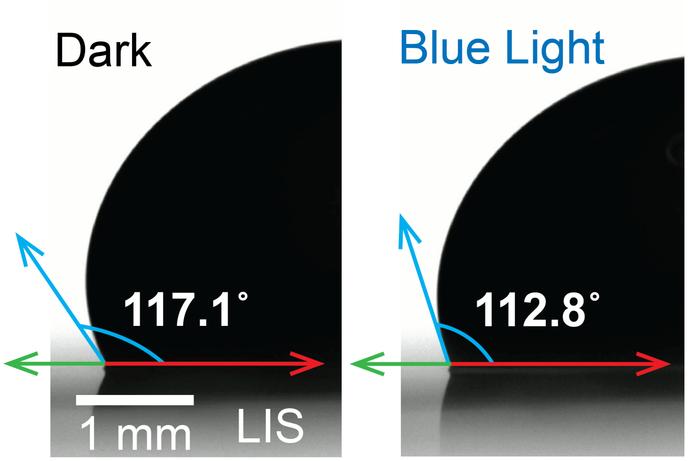

Figure S17: Contact angle measurements of 1 mM MCH-para in water at pH 3. The contact angle of MCH-para water droplet on LIS reduced from 117° to 113° upon blue light (470 nm, 31.8 mW/cm^2) irradiation.

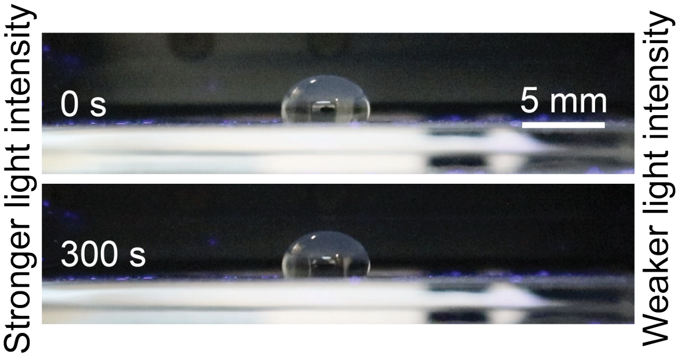

Figure S18: Optical images (side view) of a stationary pure DI water droplet illuminated by UV light with an intensity gradient after passing through an ND filter. The intensity of the light is greater on the left and weaker on the right.

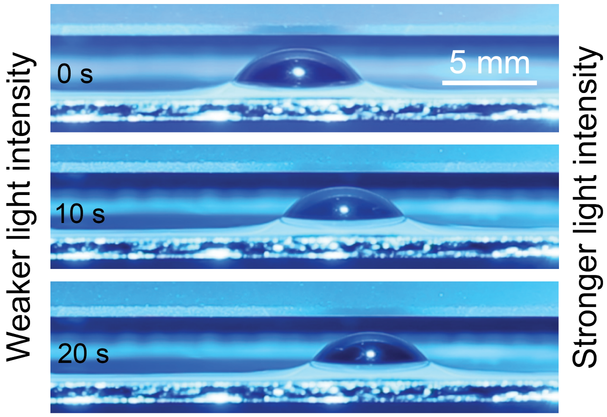

Figure S19: Time-lapse optical images (side view) of linear movement of a water droplet containing MCH-para on LIS directed by blue light with an intensity gradient.

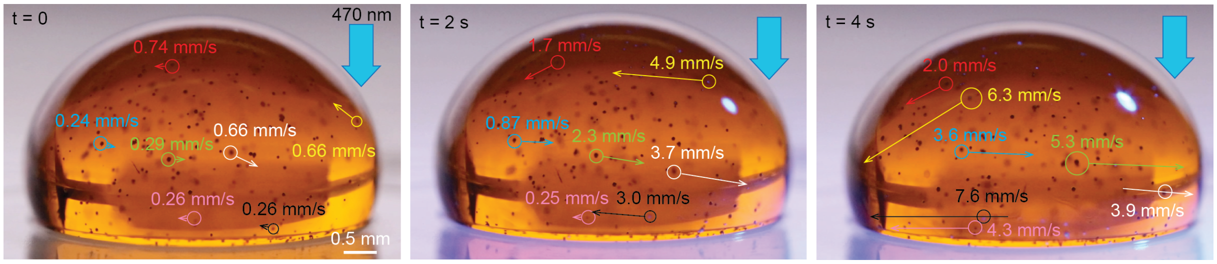

Figure S20: Time-lapse optical images (side view) of the internal flow field indicated by tracer particles of a water droplet containing MCH-para. Blue light was illuminated on the right side of the droplet. The directions and lengths of arrows indicate the directions and magnitudes of the velocity of the tracer particles.

Summarizing literature studies on droplet manipulation using light-control

Year	Results	Method	Light wave-length	Intensity of light	ΔT	Droplet velocity	Ref
**Light-induced wettability gradient on photosensitive substrate.** Altering the surface wetting properties of a light-sensitive material on substrate by exposing it to light, creating a gradient							
1) Inorganic Substrate (TiO_2, ZnO)							
1997	Wettability change of droplet	TiO_2 coated glass	UV	No data	No data	No data	(*10*)
2001	Wettability change of droplet	TiO_2 and ZnO films	UV	20 mW/cm^2	No data	No data	(*11*)
2005	Wettability change of droplet	TiO_2 nanorod films	365± 10 nm	No data	No data	No data	(*12*)
2010	Droplet motion induced by wettability change	TiO_2 nanorod in PMMA matrix	355 nm	7 mJ/cm^2	No data	No data	(*13*)
2) Photoresponsive surfactant modified surface (Azobenzene)							
2000	Droplet motion induced by wettability change	Modified surface with photoiso-merizable azobenzene	365 nm and 436 nm	1 mW/cm^2	No data	35 µm/s	(*14*)
2007	Droplet motion induced by wettability change	Modified surface with photochromic azobenzene	254 nm and 366 nm	10 mW/cm^2	No data	No data	(*15*)
**Light-induced Thermal-Marangoni effect** Generation of fluid flow and surface tension gradients in a liquid, caused by temperature differences induced by high-power light exposure							
1) Over liquid substrates							
2004	Laser-guided motion of oil droplet in water solution	I_2 and KI in oil nitrobenzene; 0.5 mM sodium dodecyl sulfate in water	532 nm	0.1-1 W (0.6 mm in diameter) I_{max}=353.7 W/cm^2	T_{max}=50 °C	V_{max}=2 cm/s	(*16*)
2) Submerged in bulk liquid							
2005	Laser-guided motion of aqueous droplets in decanol	Some additives for reaction application	1525 nm	30 mW over unspecified area	ΔT=10 °C	V_{max}=3 mm/s	(*17*)
2010	Laser-guided motion of aqueous droplets in decanol or mineral oil	0.08 mM sodium dodecyl sulfate in droplet	1457 nm IR laser	15 mW/100 µm^2 15000 W/cm^2	T_{max}=80 °C	V_{max}=1.5 µm/s	(*18*)
2011	Motion of aqueous droplets in FC-40, driven by heating from commercial computer projector	Water (with dye and Tween 20) droplets in FC-40	No data	13.53 W/cm^2	ΔT=1 °C (from simulation)	V_{max}=400 µm/s	(*19*)

**Year**	**Results**	**Method**	**Light wave-length**	**Intensity of light**	ΔT	**Droplet velocity**	**Ref**
2018	Mixture of water and ethylene glycol droplet in DCB	MUA–Au/ Fe_3O_4–OA surfactants in droplet; Toluene in DCB for density match	660 nm	70 mW 8.9 W/cm^2 (*Estimated)	T_{max}=boiling point of water and toluene	V_{max}=0.9 mm/s (flow speed)	(20)
3) Over solid substrates							
2018	Various droplets motion on photoresponsive organogel surface	Droplet: Water, glycerol, ethylene glycol, propylene glycol, ethanol Fe_3O_4 nanoparticles embedded in PDMS with lubricant	808 nm	300 mW/(1.5 × 2 mm^2) 10 W/cm^2	ΔT=56 °C	V_{max}=1.7 mm/s	(21)
2019	Laser-guided water droplet on an Fe_3O_4-doped slippery surface	Fe_3O_4 nanoparticles	808 nm	300 mW/ (2.3×1.4 mm^2) 9.3 W/cm^2	T_{max}=130 °C	V_{max}=1.15 mm/s	(22)
2022	Laser-guided aqueous droplet on lubricated surface	Droplets incorporating polypyrrole nanoparticles (PPy NPs)	808 nm	200 mW/ (0.5×2 mm^2) 100 W/cm^2	T_{max}=72 °C	V_{max}=1200 µm/s	(23)
2022	Laser-guided water droplet motion on NIR responsive slippery surface	Fabricated NIR responsive slippery surface (G/NPGS) with graphene and silicon oil	808 nm	19 W/ (2×3 mm^2) 316.7 W/cm^2	ΔT=134 °C	V_{max}=48 mm/s	(24)
2023	Laser-guided PG aqueous solution on plasma-treated indium tin oxide (ITO)-coated glass	Propylene glycol (PG) and DI water	1550 nm	323 mW (20 µm diameter) 1.03×10^5 W/cm^2	Δ T=5 °C	V_{max}=0.29 mm/s	(25)
**Light-induced Photo-Marangoni effect** The creation of fluid flow and surface tension gradients within a liquid, triggered by light exposure, which alters the chemical composition and properties of the liquid							
1) Over liquid substrates							
2009	Manipulate oil droplet floating on aqueous solution with AzoTAB	Oleic acid droplet floating on an aqueous solution; 2 mM AzoTAB in bulk aqueous	365 nm and 475 nm	No data	No data	V_{max}=300 µm/s	(26)
2014	Manipulate oil droplet floating on aqueous solution with AzoTAB	Oleic acid droplet floating on an aqueous solution; 0.1 mM AzoTAB in bulk aqueous	Mainly 365 nm 470 nm (some experi-ments)	6 W (365 nm) 11.9 W/cm^2 (*Estimated)	No data	V_{max}=500 µm/s	(27)
1) Submerged in bulk liquid							

**Year**	**Results**	**Method**	**Light wave-length**	**Intensity of light**	**ΔT**	**Droplet velocity**	**Ref**
2018	SP:HDA (2-hexyldecanoic acid) droplet on/under Water MCH^+:DBS^- (dodecylbenzene-sulfonic acid) Droplet on/under Water	SP:HDA in dichloroethane (some with toluene); MCH^+:DBS^- in nitrobenzene (some with toluene)	365 nm and 405 nm	190 mW (365 nm) over unspecified area 1 mW (405 nm)	No data	V_{max}=10.4 mm/s	(*28*)
2020	Light-guided water droplet motion in oil (ion concentration gradient)	Fenton reaction occurs on the irradiated Fe_2O_3 nanoparticles, which causes uneven ion concentration to change the surface tension	405 nm	10 mW/cm^2	No data	V_{avg}=3.1 µm/s	(*29*)
2021	Light-guided water droplet motion in organic media	$NaSO_3^-MCH^+SO_3^-$ in DI water (0.2 M); Organic media: fatty alcohols, 1-hexanol, 1-octanol, 1-decanol and cyclohexanol	365 nm and 405 nm	190 mW (365 nm); 63 mW (405 nm)	No data	V_{max}=7 mm/s	(*30*)
2022	Light-guided oil droplet immersed in aqueous solution with Azobenzene (need two lights to generate gradient)	Oil droplet produced with 4'-pentyl-4-biphenyl-carbonitrile (5CB)	365 nm and 455 nm	27.7 mW/cm^2 (UV) 3.2 mW/cm^2 (455 nm)	No data	V_{max}=40 µm/s	(*31*)
2023	Light-guided oil droplet immersed in aqueous solution with AzoTAB	Heptyloxybenzal-dehyde (HBA) droplets; Aqueous solution: 50 mM AzoTAB/DTAB (50/50 mol%) solution	365 nm 470-495 nm	1.2-3.4 mW/cm^2 (UV) 5.8-51 mW/cm^2 (visible)	No data	V_{max}=10 µm/s	(*32*)

Table S1: Summarizing literature studies on droplet manipulation using light-control.

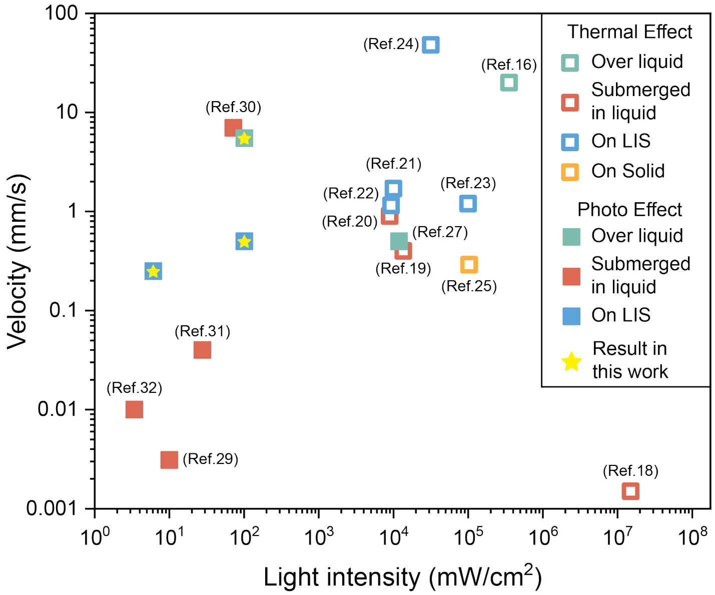

Figure S21: Summary of literature studies: correlation between light intensity and droplet velocity in droplet manipulation via thermal and photo effects.

Other supporting materials:

Movie S1: Linear movement of a water droplet containing SP-DA-PEG on LIS.

Movie S2: Water droplets containing SP-DA-PEG changing moving directions on LIS.

Movie S3: Water droplets containing SP-DA-PEG merging on LIS.

Movie S4: Internal flow field indicated by tracer particles of a water droplet containing MCH-para.

Movie S5: Linear movement and switching of the direction of a water droplet containing SP-DA-PEG on Krytox.

Movie S6: Droplet trajectory depicting a 'UCSB' pattern.

Movie S7: Toluene liquid column containing SP-DA-PEG moving inside a glass capillary tube.

References

(*1*) Wimberger, L., Prasad, S. K., Peeks, M. D., Andréasson, J., Schmidt, T. W., and Beves, J. E. (2021). Large, Tunable, and Reversible pH Changes by Merocyanine Photoacids. *Journal of the American Chemical Society 143*, 20758–20768.

(*2*) Seshadri, S., Bailey, S. J., Zhao, L., Fisher, J., Sroda, M., Chiu, M., Stricker, F., Valentine, M. T., Read de Alaniz, J., and Helgeson, M. E. (2021). Influence of Polarity Change and Photophysical Effects on Photosurfactant-Driven Wetting. *Langmuir 37*, 9939–9951.

(*3*) Zhao, L., Seshadri, S., Liang, X., Bailey, S. J., Haggmark, M., Gordon, M., Helgeson, M. E., Read de Alaniz, J., Luzzatto-Fegiz, P., and Zhu, Y. (2022). Depinning of Multiphase Fluid Using Light and Photo-Responsive Surfactants. *ACS central science 8*, 235–245.

(*4*) Aizawa, M., Namba, K., and Suzuki, S. (1977). Photo control of enzyme activity of α-amylase. *Archives of Biochemistry and Biophysics 180*, 41–48.

(*5*) Sheng, Y., Leszczynski, J., Garcia, A. A., Rosario, R., Gust, D., and Springer, J. (2004). Comprehensive theoretical study of the conversion reactions of spiropyrans: substituent and solvent effects. *The Journal of Physical Chemistry B 108*, 16233–16243.

(*6*) Klajn, R. (2014). Spiropyran-based dynamic materials. *Chemical Society Reviews 43*, 148–184.

(*7*) Kortekaas, L., Chen, J., Jacquemin, D., and Browne, W. (2018). Proton-stabilized photochemically reversible E/Z isomerization of spiropyrans. *The Journal of Physical Chemistry B 122*, 6423–6430.

(*8*) Gittens, G. (1969). Variation of surface tension of water with temperature. *Journal of Colloid and Interface Science 30*, 406–412.

(*9*) Bjelobrk, N., Girard, H.-L., Bengaluru Subramanyam, S., Kwon, H.-M., Quéré, D., and Varanasi, K. K. (2016). Thermocapillary motion on lubricant-impregnated surfaces. *Phys. Rev. Fluids 1*, 063902.

(*10*) Wang, R., Hashimoto, K., Fujishima, A., Chikuni, M., Kojima, E., Kitamura, A., Shimohigoshi, M., and Watanabe, T. (1997). Light-induced amphiphilic surfaces. *Nature 388*, 431–432.

(*11*) Sun, R.-D., Nakajima, A., Fujishima, A., Watanabe, T., and Hashimoto, K. (2001). Photoinduced surface wettability conversion of ZnO and TiO2 thin films. *The Journal of Physical Chemistry B 105*, 1984–1990.

(*12*) Feng, X., Zhai, J., and Jiang, L. (2005). The fabrication and switchable superhydrophobicity of TiO2 nanorod films. *Angewandte Chemie International Edition 44*, 5115–5118.

(*13*) Villafiorita Monteleone, F., Caputo, G., Canale, C., Cozzoli, P. D., Cingolani, R., Fragouli, D., and Athanassiou, A. (2010). Light-controlled directional liquid drop movement on TiO2 nanorods-based nanocomposite photopatterns. *Langmuir 26*, 18557–18563.

(14) Ichimura, K., Oh, S.-K., and Nakagawa, M. (2000). Light-driven motion of liquids on a photoresponsive surface. *Science 288*, 1624–1626.

(15) Yang, D., Piech, M., Bell, N. S., Gust, D., Vail, S., Garcia, A. A., Schneider, J., Park, C.-D., Hayes, M. A., and Picraux, S. (2007). Photon control of liquid motion on reversibly photoresponsive surfaces. *Langmuir 23*, 10864–10872.

(16) Rybalko, S., Magome, N., and Yoshikawa, K. (2004). Forward and backward laser-guided motion of an oil droplet. *Physical review E 70*, 046301.

(17) Kotz, K. T., Gu, Y., and Faris, G. W. (2005). Optically addressed droplet-based protein assay. *Journal of the American Chemical Society 127*, 5736–5737.

(18) Dixit, S. S., Kim, H., Vasilyev, A., Eid, A., and Faris, G. W. (2010). Light-driven formation and rupture of droplet bilayers. *Langmuir 26*, 6193–6200.

(19) Hu, W., and Ohta, A. T. (2011). Aqueous droplet manipulation by optically induced Marangoni circulation. *Microfluidics and nanofluidics 11*, 307–316.

(20) Yang, Z., Wei, J., Sobolev, Y. I., and Grzybowski, B. A. (2019). Systems of mechanized and reactive droplets powered by multi-responsive surfactants (vol 553, pg 313, 2018). *Nature 567*, E11–E11.

(21) Gao, C., Wang, L., Lin, Y., Li, J., Liu, Y., Li, X., Feng, S., and Zheng, Y. (2018). Droplets manipulated on photothermal organogel surfaces. *Advanced Functional Materials 28*, 1803072.

(22) Wu, S., Zhou, L., Chen, C., Shi, L.-A., Zhu, S., Zhang, C., Meng, D., Huang, Z., Li, J., Hu, Y., et al. (2019). Photothermal actuation of diverse liquids on an Fe3O4-doped slippery surface for electric switching and cell culture. *Langmuir 35*, 13915–13922.

(23) Hwang, H., Papadopoulos, P., Fujii, S., and Wooh, S. (2022). Driving Droplets on Liquid Repellent Surfaces via Light-Driven Marangoni Propulsion. *Advanced Functional Materials 32*, 2111311.

(24) Rao, Q., Tong, Z., Song, L., Ali, A., Hou, Y., He, Q., Lu, J., Gao, X., Zhan, X., and Zhang, Q. (2022). NIR-driven fast construction of patterned-wettability on slippery lubricant infused surface for droplet manipulation. *Chemical Engineering Journal 428*, 131141.

(25) Li, W., Li, D., Zhu, X., Ye, D., Yang, Y., Wang, H., Chen, R., and Liao, Q. (2023). Light-manipulated binary droplet transport on a high-energy surface. *Lab on a Chip 23*, 4287–4301.

(26) Diguet, A., Guillermic, R.-M., Magome, N., Saint-Jalmes, A., Chen, Y., Yoshikawa, K., and Baigl, D. (2009). Photomanipulation of a droplet by the chromocapillary effect. *Angewandte Chemie International Edition 48*, 9281–9284.

(27) Venancio-Marques, A., and Baigl, D. (2014). Digital optofluidics: LED-gated transport and fusion of microliter-sized organic droplets for chemical synthesis. *Langmuir 30*, 4207–4212.

(28) Xiao, Y., Zarghami, S., Wagner, K., Wagner, P., Gordon, K. C., Florea, L., Diamond, D., and Officer, D. L. (2018). Moving droplets in 3D using light. *Advanced Materials 30*, 1801821.

(29) Sun, D., Zhou, D., Gao, Y., Yue, H., Wang, W., Ma, X., and Li, L. (2020). Phototaxis Motion Behavior of a Self-propelled Submarine-like Water Droplet Robot in Oil Solvent. *ChemNanoMat 6*, 1611–1616.

(30) Xiao, Y., Martino, N., Wagner, K., Spinks, G. M., Officer, D. L., and Wagner, P. (2021). Photocontrolled directional transport using water-in-oil droplets. *New Journal of Chemistry 45*, 1172–1175.

(31) Ryabchun, A., Babu, D., Movilli, J., Plamont, R., Stuart, M. C., and Katsonis, N. (2022). Run-and-halt motility of droplets in response to light. *Chem 8*, 2290–2300.

(32) Kojima, T., Kitahata, H., Asakura, K., and Banno, T. (2023). Photoinduced collective motion of oil droplets and concurrent pattern formation in surfactant solution. *Cell Reports Physical Science 4*.
